# Modeling mechanical activation of macrophages during pulmonary fibrogenesis for targeted anti-fibrosis therapy

**DOI:** 10.1126/sciadv.adj9559

**Published:** 2024-03-29

**Authors:** Ying Xu, Linxuan Ying, Jennifer K. Lang, Boris Hinz, Ruogang Zhao

**Affiliations:** ^1^ Department of Biomedical Engineering, University at Buffalo, State University of New York, Buffalo, NY 14260, USA.; ^2^ Division of Cardiovascular Medicine and the Clinical and Translational Research Center, University at Buffalo, State University of New York; Veterans Affairs Western New York Health Care System, University at Buffalo, State University of New York; Department of Biomedical Engineering, University at Buffalo, State University of New York; Department of Medicine, University at Buffalo, State University of New York; Department of Pharmacology and Toxicology, University at Buffalo, State University of New York, Buffalo, NY, 14260, USA.; ^3^ Laboratory of Tissue Repair and Regeneration, Keenan Research Centre for Biomedical Science of the St. Michael’s Hospital, Toronto, ON M5B 1T8, Canada.; ^4^ Faculty of Dentistry, University of Toronto, Toronto, ON, Canada.

## Abstract

Pulmonary fibrosis is an often fatal lung disease. Immune cells such as macrophages were shown to accumulate in the fibrotic lung, but their contribution to the fibrosis development is unclear. To recapitulate the involvement of macrophages in the development of pulmonary fibrosis, we developed a fibrotic microtissue model with cocultured human macrophages and fibroblasts. We show that profibrotic macrophages seeded on topographically controlled stromal tissues became mechanically activated. The resulting co-alignment of macrophages, collagen fibers, and fibroblasts promoted widespread fibrogenesis in micro-engineered lung tissues. Anti-fibrosis treatment using pirfenidone disrupts the polarization and mechanical activation of profibrotic macrophages, leading to fibrosis inhibition. Pirfenidone inhibits the mechanical activation of macrophages by suppressing integrin αMβ2 and Rho-associated kinase 2. These results demonstrate a potential pulmonary fibrogenesis mechanism at the tissue level contributed by macrophages. The cocultured microtissue model is a powerful tool to study the immune–stromal cell interactions and the anti-fibrosis drug mechanism.

## Teaser

Engineered lung microtissue was used to study macrophage-induced lung fibrosis development and anti-fibrosis treatment.

## INTRODUCTION

Pulmonary fibrosis, as seen in idiopathic pulmonary fibrosis (IPF) and COVID-induced pulmonary fibrosis, is a serious and often fatal lung disease (*1*, *2*). Pulmonary fibrosis is characterized by the substantial changes in the architecture, composition, and stiffness of the lung tissue that lead to the deterioration of lung functions (*3*). Pulmonary fibrosis initiates as a result of alveolar epithelial injury, followed by the recruitment and activation of immune cells and ensuing aberrant wound healing (*4*). Increased accumulation of macrophages, including unique pro-fibrotic subpopulations, has been reported in the fibrotic lungs (*5*). However, the contribution of different macrophage activation states to the disease progression and severity is still unclear (*6*). For instance, macrophages can either contribute to normal repair or attain pro-fibrotic roles, depending on timing, context, and their activation status (*7*, *8*). The absence of macrophages during inflammation has been shown to result in impaired wound healing (*9*, *10*), while macrophage persistence beyond acute repair phases can result in organ fibrosis (*11*–*13*). Recent studies suggest that the latter effect is at least in part due to the contribution of pro-fibrotic macrophages to the activation of fibroblasts into to myofibroblasts in a process that depends on physical contact (*14*, *15*). Myofibroblasts are main effector cells in fibrogenesis by producing excessive amounts of collagen and generating high contractile forces by incorporating α-smooth muscle actin (α-SMA) into stress fibers (*16*). However, it is still unclear how macrophage-myofibroblast communication at the tissue level contributes to lung tissue fibrogenesis.

In the fibrotic lung tissue, the excessively accumulated fibrillar collagen is often aligned in dense bundles, as a result of the active extracellular matrix (ECM) remodeling by contractile myofibroblasts (*17*). This pathologically stiff tissue microenvironment also provides topographic cues to the embedded cells, causing changes in the cellular functions such as their morphology and alignment (*18*, *19*). For instance, bone marrow–derived macrophages have been shown to align in vitro with engineered topographic cues such as microgrooves produced in 2D elastic substrates; alignment promotes profibrotic polarization into pro-fibrotic macrophages characterized by expression of arginase-1 and CD206 (*20*). For simplicity, in vitro–generated CD206-positive macrophages are often called M2 phenotypes, recognizing that macrophages exist in a more complex polarization (activation) spectrum in vivo (*5*, *14*, *21*–*25*). Whether and how mechanical and topographical properties of fibrotic tissue microenvironment affect macrophage activation into different polarization states and whether macrophage mechanosensing plays a role in the progression of fibrosis are largely unknown.

Their involvement in fibrogenesis makes immune cells attractive targets for anti-fibrosis therapies (*26*). Pirfenidone (PFD) is a Food and Drug Administration (FDA)–approved anti-fibrotic drug known to act on multiple fibrogenic pathways (*27*). PFD reduces fibroblast proliferation and inhibits transforming growth factor–β (TGF-β) pathway (*28*). Recent studies have shown that PFD can also modulate the polarization and fibrogenic activities of macrophages (*29*, *30*), but because these studies were performed in animal models, it is unknown whether such findings can be applied to humans. Lack of human-relevant, disease-mimetic research models has been one of the major barriers in the development of anti-fibrosis drugs.

Through histological and single-cell sequencing analyses of human fibrotic lung samples, we here show that profibrotic macrophages are mechanically activated in fibrotic lung microenvironment. We developed fibrotic microtissues with cocultured human macrophage and lung fibroblasts to model mechanical activation of macrophages and subsequent fibrogenesis. Fibroblast-populated, membranous lung microtissues were first fabricated on a group of micropillars to guide the alignment of collagen fibers and fibroblasts. Profibrotic macrophages seeded onto this tissue became mechanically activated, and their extensive co-alignment with collagen fibers and fibroblasts promoted widespread fibrogenesis in the lung microtissue. PFD treatments disrupted the polarization and mechanical activation of M2 macrophages, leading to fibrosis inhibition. We showed that PFD inhibited macrophage mechanical activation by suppressing integrin αMβ2 (CD11b/CD18) and Rho-associated kinase 2 (ROCK2), which is a previously unknown mechanism of action of PFD. Together, these results reveal a previously unidentified mechanism by which mechanically activated macrophages contribute to pulmonary fibrogenesis at the tissue level. The heterocellular force-sensing microtissue model is shown as a powerful tool to study the complex immune–stromal cell interactions and the mechanism of action of anti-fibrosis drugs.

## RESULTS

### Lung macrophages undergo mechanical activation in fibroblast-remodeled fibrotic tissue microenvironment

**Fig. 1.**
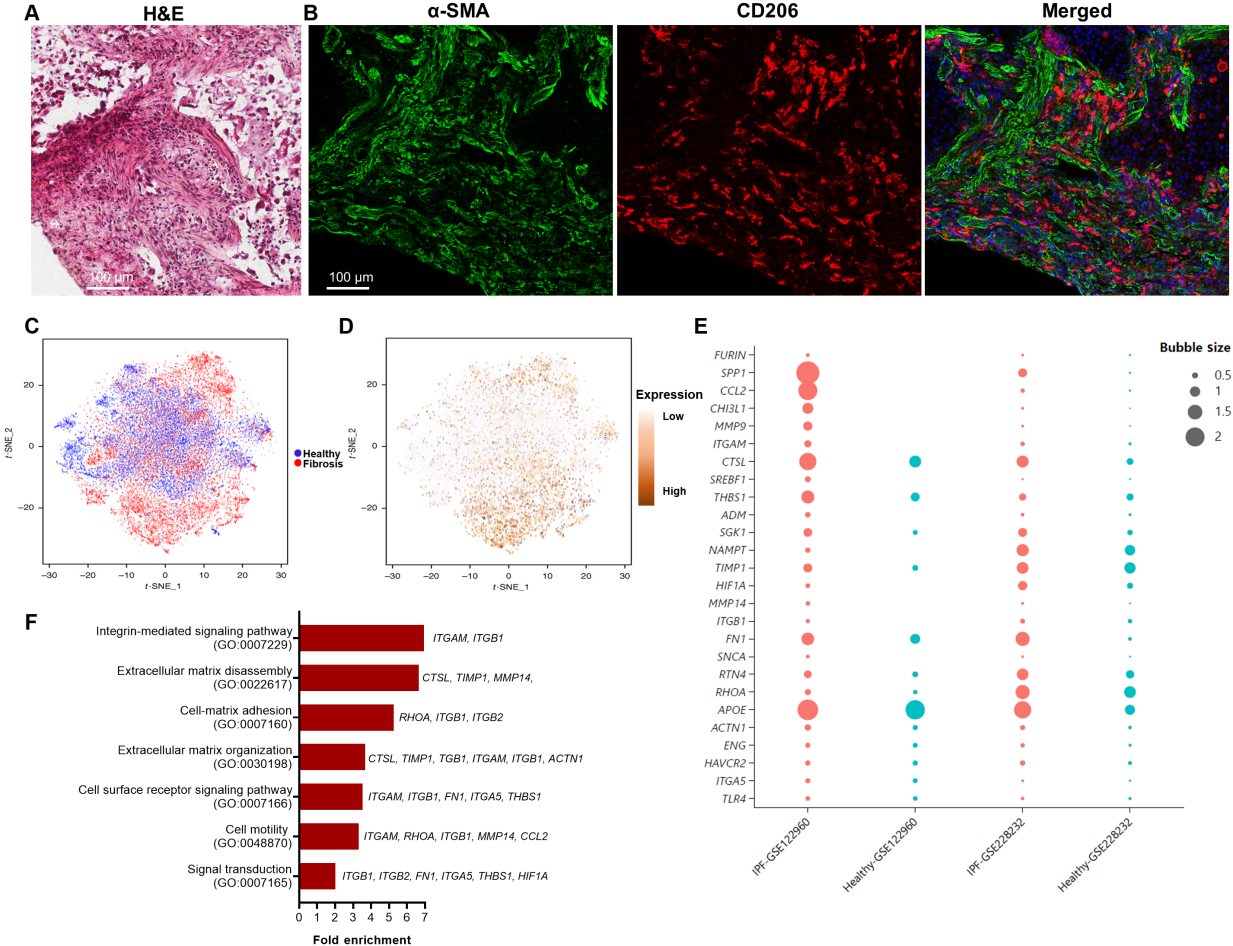
Mechanical activation of lung macrophages in a highly remodeled fibrotic tissue microenvironment. (**A**) Hematoxylin and eosin (H&E) staining of a highly remodeled region in a fibrotic lung tissue. (**B**) Confocal fluorescence images of α-SMA and CD206 of a matching region in an adjacent tissue section. Both α-SMA^+^ myofibroblasts and CD206^+^ macrophages adopted elongated morphology and aligned with each other. Scale bars, 100 μm. (**C**) *t*-Distributed stochastic neighbor embedding (*t*-SNE) plot showing the distinct populations of alveolar macrophages in patients with IPF and healthy donors. (**D**) *t*-SNE plot showing the preferential expression of *ITGAM* gene in alveolar macrophages in the patients with IPF illustrated in (C). Data extracted from healthy subjects (*n* = 8) and patients with IPF (*n* = 4) based on NCBI GEO dataset GSE122960. (**E**) Expression of genes linked to the activation of mechanobiological pathways in alveolar macrophages of healthy donors (blue) and patients with IPF (orange). Bubble size corresponds to the average expression level of the gene within each group. The data were extracted from the NCBI GEO datasets GSE122960 and GSE228232. (**F**) Fold enrichment of Gene Ontology (GO) biological process in genes that are up-regulated in fibrotic lungs compared to healthy donor lungs. The GO enrichment data were collected from dataset GSE122960.

To spatially resolve the interaction of collagen, macrophages, and fibroblasts in human fibrotic lungs, we processed lung tissues from donors suffering from IPF for histological analysis. Hematoxylin and eosin (H&E) staining of highly fibrotic regions revealed a significantly remodeled tissue architecture, characterized by large amounts of elongated cells embedded between dense and aligned ECM fibers (Fig. 1A and fig. S1). The embedded cells consisted of a large number of α-SMA–positive myofibroblasts and CD206-positive macrophages that were in proximity. Myofibroblasts and macrophages both adopted elongated morphologies and aligned with each other, suggesting that they sensed and responded to the remodeled tissue microenvironment (Fig. 1B and fig. S1). A reanalysis of the dataset from a single-cell RNA sequencing (scRNA-seq) study of human lung samples uncovered a distinctive subpopulation of profibrotic alveolar macrophages exclusive to patients with pulmonary fibrosis (Fig. 1C) (*31*). Notably, Integrin subunit Alpha M (*ITGAM*), which encodes integrin αM and plays important roles in cell adhesion and mechanotransduction, was preferentially expressed in macrophages within the IPF group (Fig. 1D) (*31*). When a comprehensive list of 224 genes associated with mechanotransduction pathways was cross-referenced with National Center for Biotechnology Information (NCBI) Gene Expression Omnibus (GEO) datasets GSE122960 and GSE228232 (*5*, *31*), 28 genes were identified as up-regulated in macrophages from patients with IPF compared to the healthy donors (Fig. 1E). Functional enrichment analysis of these genes using Gene Ontology (GO) biological processes shows that the up-regulation of these genes is strongly associated with the activation of mechanobiological processes such as cell-matrix adhesion, integrin-mediated signaling and cell motility (Fig. 1F) (*31*). Together, these analyses suggest that lung macrophages undergo mechanical activation within the extensively remodeled fibrotic tissue microenvironment of human IPF lungs.

### Macrophages are mechanically responsive to topography-controlled tissue remodeling

**Fig. 2.**
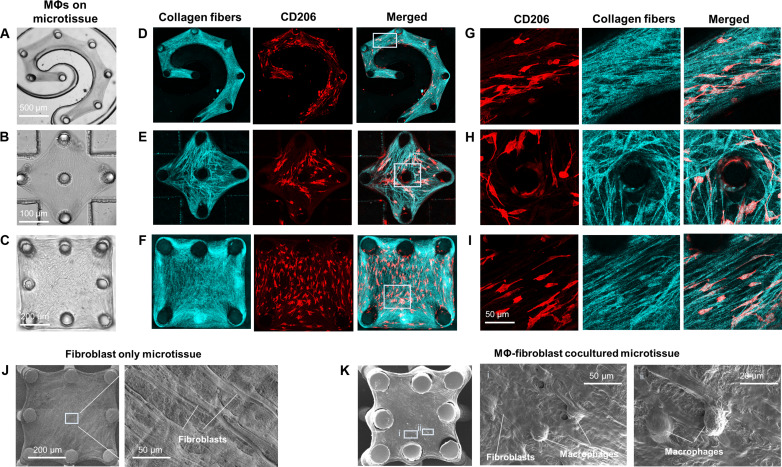
Macrophage mechanical activation in response to topographically controlled tissue remodeling. Phase contrast images of fibroblast and macrophage cocultured lung microtissues formed on a group of flexible micropillars arranged in a spiral pattern (**A**), a diamond pattern (**B**), and a square pattern (**C**). Confocal reflectance images of collagen fibers (cyan) and fluorescence images of CD206 (red) of spiral patterned (**D**), diamond patterned (**E**), and square patterned (**F**) microtissues. (**G** to **I**) Enlarged views of boxed regions in (D) to (F) showing the co-alignment between the CD206-positive macrophages and the collagen fibers. (**J**) Scanning electron microscopy (SEM) images of a fibroblast only microtissue showing the fibroblast alignment. (**K**) SEM images of a cocultured microtissue showing the co-alignment between fibroblasts and macrophages.

To understand how macrophages respond to remodeled tissue architecture such as those seen in fibrotic lung tissue, we created engineered microtissues with controlled morphology and cell and ECM alignment. Normal human lung fibroblasts (NHLFs) were mixed with collagen type I and seeded into microfabricated microwells containing a group of micropillars arranged in topological patterns. Pattern designs included a spiral pattern (Fig. 2A), diamond pattern (Fig. 2B), and square pattern (Fig. 2C) to mimic various geometries found in the lung tissue such as highly curved alveolar wall. Fibroblasts generated contractile forces and compacted the collagen ECM against the micropillars. As a result of fibroblast-mediated ECM remodeling, morphologically controlled microtissues formed following the micropillar array pattern templates. NHLF and collagen fibers in the microtissues were strongly co-aligned along the lines of tension defined by the micropillar boundary conditions (fig. S3). Thus, controlled cell and ECM fiber alignment provide an opportunity to examine macrophage response to the tissue topography observed in fibrosis. Next, monocytes derived from human peripheral blood were pre-polarized with interleukin-4 (IL-4) and IL-13 before being seeded onto NHLF-remodeled microtissues. Such M2 macrophages adhered to the microtissue surface within 48 hours and adopted an elongated spindle morphology aligning with collagen fibers and NHLF (Fig. 2, D to F, J, and K, and fig. S3). Confocal imaging revealed that macrophages at least partially penetrated the microtissue and interacted with the surrounding collagen fibers and NHLF in three dimensions, as demonstrated by the overlapping collagen fibers and macrophages (Fig. 2, G to I). Strong expression of integrin β2 (CD18) was detected in elongated macrophages that aligned along the highly curved edge of the spiral-shaped microtissue (fig. S3), suggesting that the engagement between macrophages and the surrounding collagen fibers and NHLFs involves mechanotransduction. These findings imply that M2 macrophages have the ability to perceive topographic cues in fibrosis-like tissue microenvironments and dynamically adjust their mechanotransduction pathways in response to these cues.

### Extensive co-alignment between macrophages and fibroblasts promotes a widespread fibrosis response in engineered microtissues

**Fig. 3.**
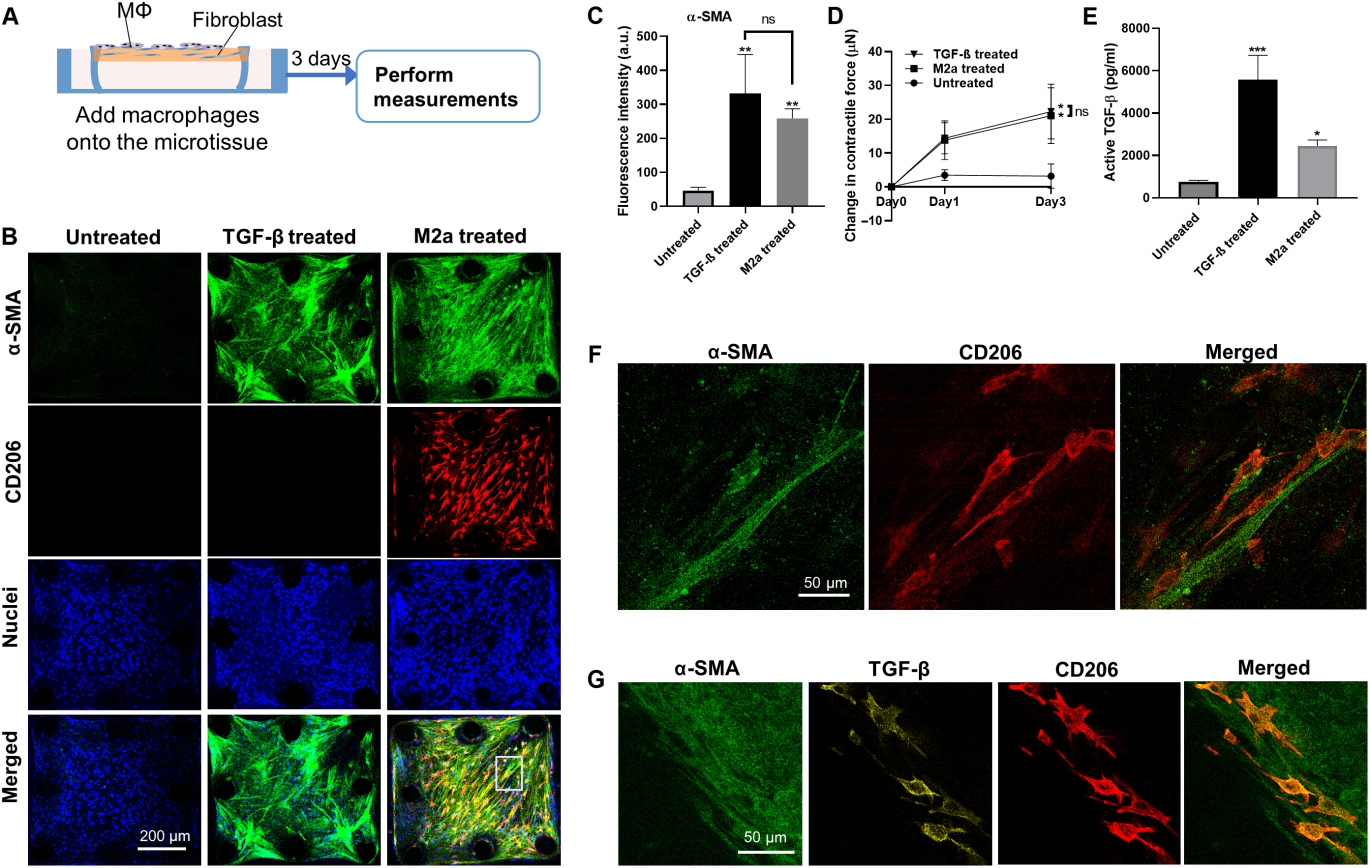
Extensive co-alignment between macrophages and fibroblasts promoted a widespread fibrosis response in the microtissue. (**A**) Schematic showing the fibrosis induction in NHLF-populated microtissues by pro-fibrotic macrophages. (**B**) Representative fluorescence images of NHLF only (untreated), TGF-β1–treated NHLF only, and M2 macrophage and NHLF cocultured microtissues (left to right). Microtissues were stained for nuclei (blue), α-SMA (green) and CD206 (red). Scale bar, 200 μm. (**C**) Fluorescence intensity measurement of α-SMA for different microtissue culture conditions. a.u., arbitrary units; ns, not significant. (**D**) Contractile force measurement for different microtissue culture conditions. (**E**) Supernatant active TGF-β level for different microtissue culture conditions. (**F**) Enlarged view of the boxed region in (B) showing the co-alignment between single myofibroblasts and M2 macrophages. (**G**) Enlarged view shows positive TGF-β signal co-localizing with CD206 but not with α-SMA. *P ≤ 0.05, **P ≤ 0.01, and ***P ≤ 0.001 when compared to untreated group by one-way ANOVA with Tukey test; 'ns' indicates no significance between two treatment groups using a two-tailed paired Student's *t *test.

Next, we sought to understand how profibrotic macrophages contribute to the formation of fibrosis in the macrophage-NHLF coculture microtissues. Profibrotic M2 macrophages were introduced to the surface of NHLF-populated microtissues (Fig. 3A). After 3 days of coculture, robust expression of α-SMA was observed in the microtissues, indicating NHLF activation into myofibroblasts (Fig. 3B). The expression levels of α-SMA in M2 macrophage cocultured microtissues were significantly higher (4.7-fold) than those in NHLF-only microtissue but moderately lower than TGF-β1–treated positive controls (Fig. 3, B and C). In contrast, monocytes introduced to NHLF-populated microtissues failed to induce positive α-SMA expression (fig. S4), suggesting that they lack the ability to induce fibrotic differentiation of the microtissue. Because differentiated myofibroblasts have been shown to generate high contractile forces, we measured the contractile force generation in monocultured and cocultured microtissues using in situ micropillar force sensors. M2 macrophage and NHLF cocultured microtissue was shown to generate 5.7 times higher contractile force as compared to NHLF monocultured microtissues (Fig. 3D). When monocytes or macrophages were seeded individually with collagen matrix, they failed to compact the collagen and form the microtissue (fig. S5), which suggests a lack of contractile force generation in these cells. Similar to the contractile force measurement, the level of active TGF-β measured in the macrophage-fibroblast cocultured microtissue supernatant was higher than that of NHLF monocultured microtissue (Fig. 3E), suggesting a potential role of paracrine TGF-β signaling in macrophage-mediated tissue fibrosis.

Because early studies have shown that activation of fibroblasts by macrophages mainly occurs in close proximity (*15*), we examined co-registration of both cells in the cocultured microtissue. Corresponding to the patterns observed in fibrotic lung tissues (Fig. 1A and fig. S1), elongated CD206-positive M2 macrophages abundantly aligned with α-SMA–positive myofibroblasts throughout the whole microtissue (Fig. 3B, merged). At high microscopy resolution, single macrophages were in proximity and/or direct contact with myofibroblasts (Fig. 3F). Immunostaining of TGF-β showed positive TGF-β signal co-localizing with CD206 but not with α-SMA, suggesting that these TGF-β were secreted by the macrophages and deposited into the ECM (Fig. 3G and fig. S6). Our previous study has shown that ECM-stored latent TGF-β can be activated locally by fibroblasts for myofibroblast differentiation (*15*). To test whether close proximity of macrophages is required for TGF-β activation by fibroblasts, we performed a condition medium transfer assay. M2 macrophage-conditioned medium applied to NHLFs did not significantly induce α-SMA expression, but heat-activated M2 macrophage conditioned medium significantly induced α-SMA expression in NHLFs (fig. S7). The different results from naive macrophage medium (without heat treatment) and heat-activated medium show that latent TGF-β in naive conditioned medium has minimal impact on myofibroblast differentiation, and it needs to be activated either by heat treatment or by fibroblasts through local integrin engagement for fibrosis induction.

### Anti-fibrosis treatment with PFD acts on macrophage-induced fibrosis

PFD and Nintedanib are the only FDA-approved drugs for the treatment of lung fibrosis; both with unclear modes of action and cellular targets in the fibrotic tissue (*32*). Our macrophage-NHLF cocultured microtissue (MaFiCo) is an excellent fibrosis model to interrogate the action of anti-fibrotic drugs. As a paradigm drug, we applied PFD to our microtissue model using (i) preventative treatment, (ii) therapeutic treatment, and (iii) pretreatment. The purpose of these different regimens was to work either without preexisting fibrosis condition (preventative treatment) or with preexisting fibrosis condition (therapeutic treatment). Pretreatment was performed only on macrophages to decouple PFD’s effect on the two cell types. PFD was examined at concentrations of 10 and 1000 μg/ml. It is noteworthy that the serum PFD concentration in the clinical setting is around 10 μg/ml (*33*).

**Fig. 4.**
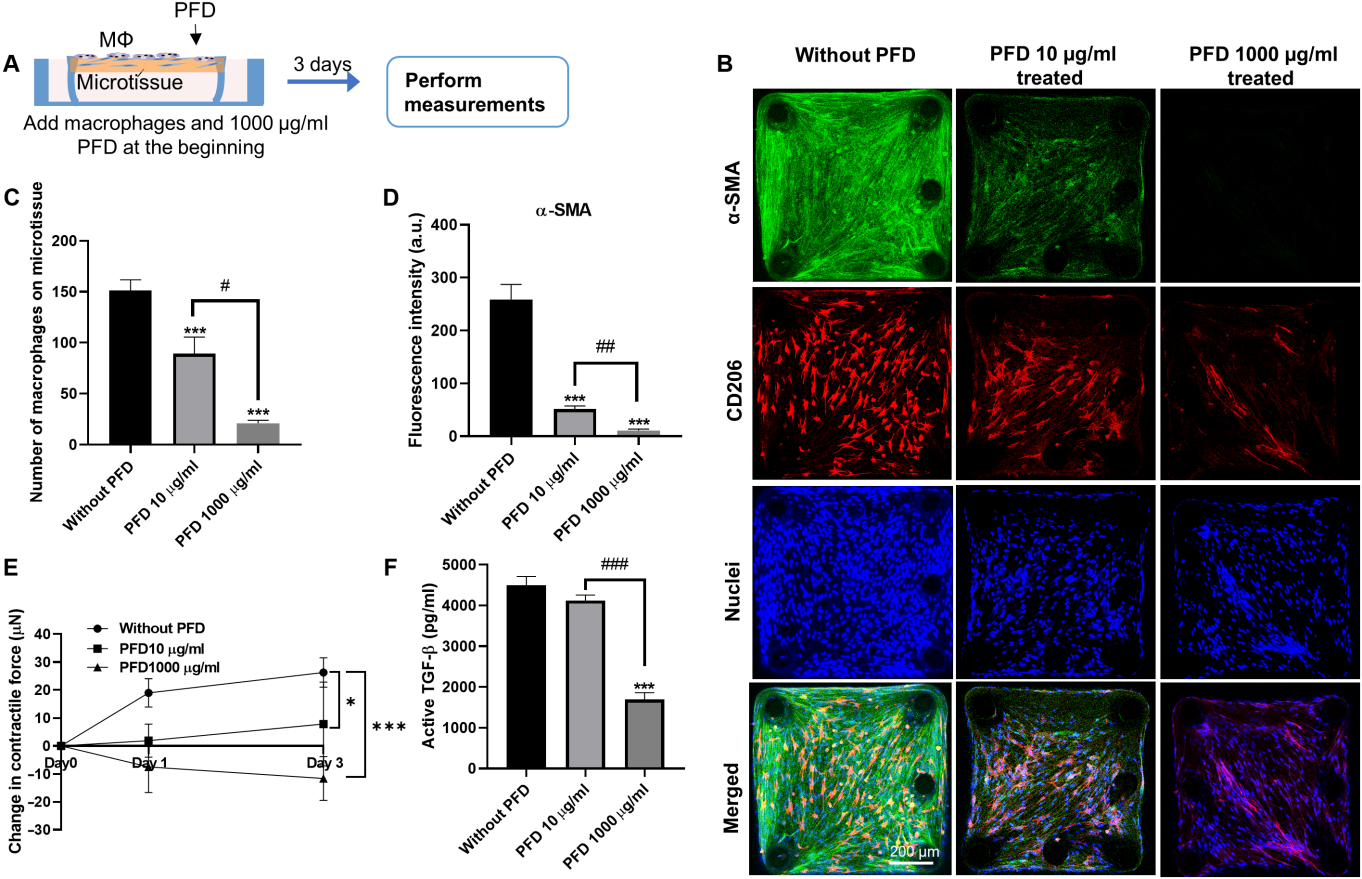
The effect of preventative anti-fibrosis treatment on cocultured microtissue. (**A**) Schematic showing the preventative anti-fibrosis treatment and results evaluation. PFD was administrated at the beginning of the coculture. (**B**) Representative fluorescence images of cocultured microtissues treated with PFD (0, 10, or 1000 μg/ml) (left to right). Scale bar, 200 μm. (**C**) The number of macrophages adhered on the microtissue under different PFD treatments. (**D**) Fluorescence intensity measurement of α-SMA under different PFD treatment conditions. (**E**) Contractile force measurement under different PFD treatment conditions. (**F**) Supernatant active TGF-β level under different PFD treatment conditions. *P ≤ 0.05 and ***P ≤ 0.001 when compared with untreated group by one-way ANOVA with Tukey test; #*P* ≤ 0.05, ##P ≤ 0.01, and ###*P* ≤ 0.001 when comparison were performed between treatment groups using a two-tailed paired Student's t test.

In the preventative treatment, PFD was introduced together with the macrophages to the NHLF-populated microtissues and maintained in the coculture for 3 days (Fig. 4A). Compared to untreated controls, preventative PFD treatment significantly reduced the number of adhered macrophages by 50 and 86% (Fig. 4, B and C), α-SMA expression in NHLFs by 80 and 95% (Fig. 4, B and D), and microtissue contractile forces by 90 and 144% (Fig. 4E) at a concentration of 10 and 1000 μg/ml, respectively. PFD at 10 μg/ml did not significantly inhibit the active TGF-β in the culture supernatant but reduced active TGF-β level by 62% at a concentration of 1000 μg/ml (Fig. 4F). These results show that preventative PFD treatment at clinically relevant concentration (10 μg/ml) is already effective in inhibiting several fibrosis promoting factors. At the highest concentration of 1000 μg/ml, PFD almost entirely abolished macrophage adhesion to the microtissue surface, the expression of α-SMA in NHLF (Fig. 4B), the deposited TGF-β in the ECM (fig. S6), and the expression of fibronectin in the microtissue (fig. S8). The few remaining macrophages in this condition exhibited super elongated shapes, which were markedly different from the spindle shapes attained in untreated controls (Fig. 4B). Because the microtissues did not have preexisting fibrotic features in the preventative treatment, these results suggest that disruption of macrophage adhesion to the microtissues inhibited the development of fibrosis.

**Fig. 5.**
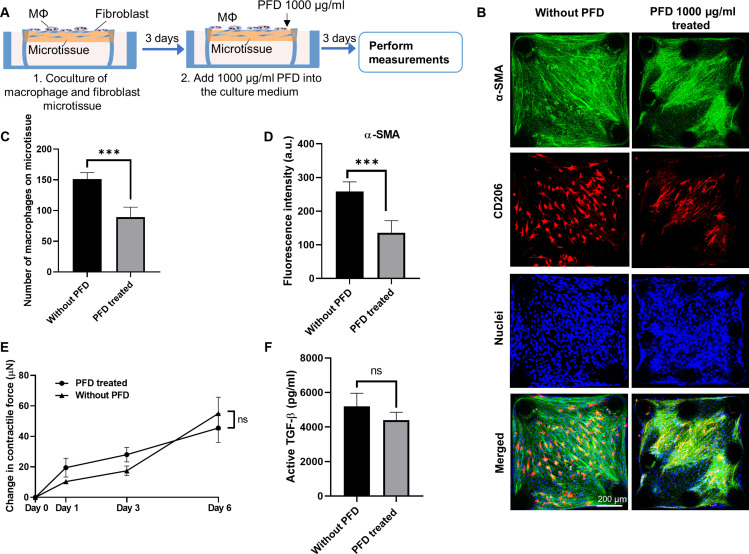
The effect of therapeutic anti-fibrosis treatment on cocultured microtissue. (**A**) Schematic showing the therapeutic anti-fibrosis treatment and results evaluation. PFD was administrated after 3 days of coculture. (**B**) Representative fluorescence images of cocultured microtissues treated with PFD (1000 μg/ml). Scale bar, 200 μm. (**C**) The number of macrophages adhered on the microtissue under different PFD conditions. (**D**) Fluorescence intensity measurement of α-SMA under different PFD treatment conditions. (**E**) Contractile force measurement under different PFD treatment conditions. (**F**) Supernatant active TGF-β level under different PFD treatment conditions. ***P ≤ 0.001 when compared with untreated group by one-way ANOVA with Tukey test; 'ns' indicates no significance between two treatment groups using a two-tailed paired Student's *t *test.

In the therapeutic treatment regimen, macrophages and NHLF were cocultured for 3 days to form fibrotic microtissues before PFD was introduced for another 3 days (Fig. 5A). PFD treatment only moderately reduced the numbers of attached macrophages (40%) (Fig. 5, B and C), α-SMA expression in NHLFs (47.5%) (Fig. 5, B and D), and levels of active TGF-β in the supernatant (Fig. 5F), as compared to the untreated control microtissues. The change in the microtissue contractile forces under PFD treatment is not significant (Fig. 5E). Furthermore, PFD treatment caused a significant change in the morphology of adhered macrophages from spindle to thread shapes (Fig. 5B), as seen in the preventative treatment. However, compared to the preventative treatment (Fig. 4C), more macrophages remained adherent in the therapeutic treatment (Fig. 5C).

**Fig. 6.**
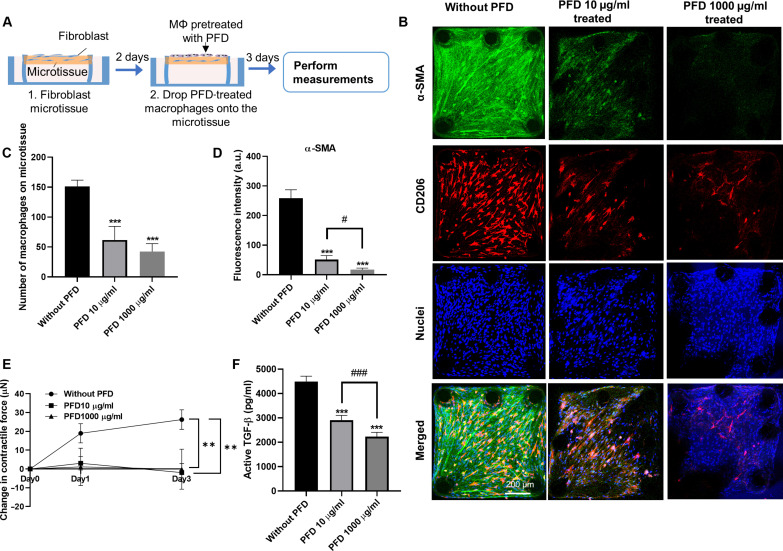
The effect of anti-fibrosis pretreatment on cocultured microtissue. (**A**) Schematic showing the anti-fibrosis pretreatment and results evaluation. Macrophages were pretreated with PFD for 24 hours and then added to NHLF microtissues to form the coculture. (**B**) Representative fluorescence images of cocultured microtissues treated with PFD (0, 10 or 1000 μg/ml) (left to right). Scale bar, 200 μm. (**C**) The number of macrophages adhered on the microtissue under different PFD conditions. (**D**) Fluorescence intensity measurement of α-SMA under different PFD treatment conditions. (**E**) Contractile force measurement under different PFD treatment conditions. (**F**) Supernatant active TGF-β level under different PFD treatment conditions. **P ≤ 0.01, and ***P ≤ 0.001 when compared with untreated group by one-way ANOVA with Tukey test; #*P *≤ 0.05 and ###*P *≤ 0.001 when comparison were performed between treatment groups using a two-tailed paired Student's *t* test.

Because PFD treatment affected both macrophages and NHLF, we next pretreated macrophages with PFD to decouple its effect on these two cell types. Human peripheral blood–derived monocytes were first treated with macrophage colony-stimulating factor (M-CSF), followed by addition of PFD for 24 hours before inducing M2 polarization with IL-4/IL-13 for another 24 hours (Fig. 6A). Adding PFD during induced M2 polarization inhibited the expression of CD206 and reduced active TGF-β levels in the macrophage monoculture supernatant in a dose-dependent manner, without compromising cell viability or adhesion on the plastic dish (fig. S10). Such pretreated macrophages were then seeded onto NHLF-populated microtissues and maintained without further addition of PFD. After a 3-day coculture, there were significant reductions in the number of adhered macrophages by 59 and 72% (Fig. 6, B and C), α-SMA expression in NHLFs by 80 and 93% (Fig. 6, B and D), microtissue contractile forces by both 100% (Fig. 6E), and levels of active TGF-β by 35 and 50% (Fig. 6F) for PFD pretreatment of 10 and 1000 μg/ml, respectively. Pretreatment with PFD at 1000 μg/ml also significantly reduced deposited TGF-β in the ECM (fig. S6) and fibronectin expression in the microtissue (fig. S8). Because substantial macrophage loss was observed on the microtissue model, we seeded pretreated macrophages on physiologically soft poly(dimethylsiloxane) (PDMS) substrate (stiffness of 1 kPa) to understand the effect of substrate stiffness on macrophage adhesion. The adhesion of PFD pretreated macrophages was completed abolished on physiologically soft substrate (fig. S11), suggesting that the tissue-like soft substrate amplified the detrimental effect of PFD treatment on the macrophage adhesion. Together, these results showed that PFD inhibits both induced M2 polarization of the macrophages and their capability to adhere to NHLF-populated microtissues. Because both functions are related to macrophage-mediated fibrogenesis, PFD may act on macrophages to affect their contribution to the fibrosis process.

### PFD suppresses mechanical activation of macrophages through inhibition of ROCK2 and integrin αMβ2

**Fig. 7.**
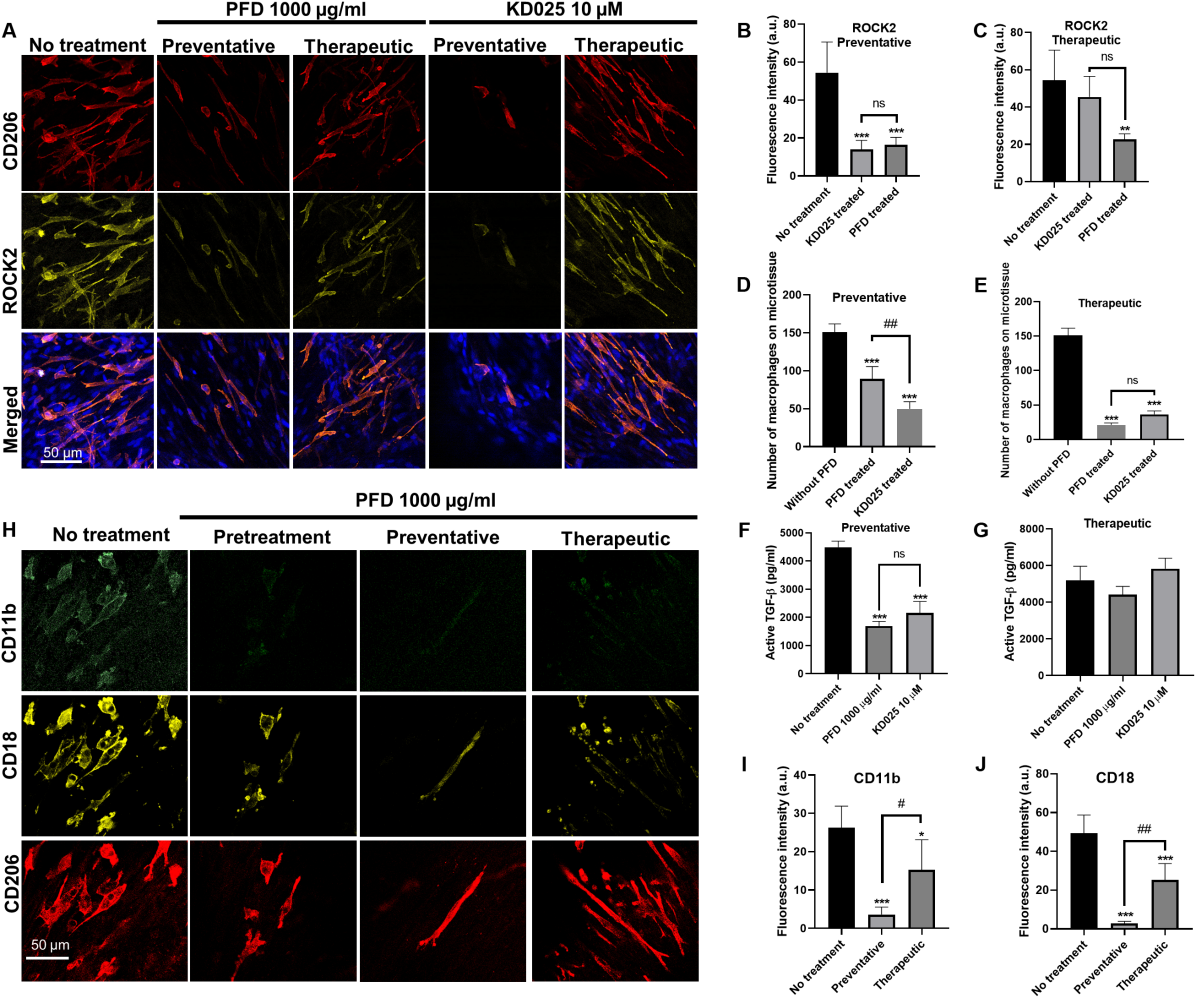
PFD suppresses macrophage mechanical activation through inhibition of ROCK2 and integrin αMβ2. (**A**) Representative immunofluorescence images of macrophage and fibroblast cocultured microtissues under PFD or ROCK2 inhibitor KD025 treatments. Microtissues were stained for CD206 (red), ROCK2 (yellow), and nuclei (blue) from top to bottom. Fluorescence intensity measurement of ROCK2 in cocultured microtissues under preventative treatment (**B**) and therapeutic treatment (**C**). Number of adherent macrophages on cocultured microtissues under preventative treatment (**D**) and therapeutic treatment (**E**). Active TGF-β in cell culture supernatant under preventative treatment (**F**) and therapeutic treatment (**G**). (**H**) Representative immunofluorescence images of macrophage and fibroblast cocultured microtissues under different PFD treatment conditions. Microtissues were stained for CD11b (green), CD18 (yellow), and CD206 (red) from top to bottom. Fluorescence intensity measurement of CD11b (**I**) and CD18 (**J**) under treatments with or without PFD. *P ≤ 0.05, **P ≤ 0.01, and ***P ≤ 0.001 when compared with untreated group by one-way ANOVA with Tukey test; 'ns' indicates no significance between two treatment groups using a two-tailed paired Student's t-test; #*P *≤ 0.05 and ##*P *≤ 0.01 between two treatment groups using a two-tailed paired Student's *t *test.

Treatment with PFD reduced the ability of macrophages to attach, align, and spread on microtissues, which are all cell functions dependent on cell adhesion. In turn, M2 polarization has been shown to require strong adhesion and intracellular cytoskeleton reorganization (*34*). Concomitantly, we identified up-regulated genes associated with the mechanotransduction processes such as cell-matrix adhesion, integrin-mediated signaling, and cell motility in published scRNA-seq datasets of lung macrophages of patients with IPF (Fig. 1) (*31*). Among these genes, ras homolog family member A (*RHOA*), *ITGAM*, and are highly up-regulated genes that are closely related to cell-matrix adhesion and integrin-mediated signaling. Thus, we focused on RhoA-related pathways, αM integrin, and β2 integrin to examine whether PFD suppresses fibrogenesis by acting on macrophage mechanosensing and activation. Rho-associated kinases ROCK1 and ROCK2 are major downstream effectors of the RhoA, and ROCK2 has been used as a therapeutic target in clinical trials of anti-fibrosis drug KD025 (*35*). Immunostaining of ROCK2 in the coculture microtissues shows that ROCK2 predominantly expressed in the M2 macrophages. Treatment with PFD caused 65 and 74% reduction in the expression of the ROCK2 in both preventative and therapeutic treatment regimens (Fig. 7, A to C). Next, we treated the coculture microtissue with KD025, a ROCK2-specific inhibitor, to determine whether the suppression of macrophage mechanical activation is ROCK2-specific. KD025 was shown to cause 39 and 21% reductions in the expression of the ROCK2 in preventative and therapeutic treatments, respectively (Fig. 7, A to C), together with significant reduction in the adhered macrophage numbers (75% in preventative and 67% in therapeutic) (Fig. 7, D and E), significant change in the macrophage morphology from spindle to thread shape and significant reduction in the active TGF-β (62% in preventative and 15% in therapeutic) (Fig. 7, F and G). These effects are very similar to those of the PFD treatment. To determine whether the above effects are specific to the macrophages, both PFD and KD025 treatment were performed on monoculture M2 macrophages on silicone substrate (stiffness of 4 MPa). ROCK2 expression was significantly reduced by PFD treatment (71%) and KD025 treatment (70%), together with 41 and 67% reductions in the number of attached macrophages and 40 and 35% reductions in the active TGF-β (fig. S12). These effects are similar to those in the coculture, suggesting that the inhibition of ROCK2 pathway is specific to the M2 macrophages.

We next examined the expression of αM and β2 integrins in coculture microtissues. Immunostaining showed that these integrins exclusively expressed in the M2 macrophages. Treatment with PFD caused 64 and 83% reductions in the expression of the αM and β2 integrin subunits in both preventative and pretreatment regimens (Fig. 7, H and I). To determine whether this effect is specific to the macrophages, PFD treatment was performed on M2 macrophages in monocultures. Expression of αM and β2 integrins was also significantly reduced by PFD treatment in the monoculture (65 and 96%) (fig. S13). Because RhoA/ROCK signaling affects the expression of integrins, we also treated the M2 macrophage monoculture using KD025. ROCK2 inhibition by KD025 significantly reduced expression of the αM (81%) and β2 (60%) integrins (fig. S13), similar to the effect of PFD treatment. Together, these results show that PFD has inhibitory effects on the macrophage mechanotransduction pathways including the ROCK2 and αM and β2 integrins, and these effects are similar to those of the selective ROCK2 inhibitor KD025.

## DISCUSSION

Pulmonary fibrosis is a deadly disease, but its disease mechanism is still unclear. The involvement of the M2 profibrotic macrophages in the pulmonary fibrogenesis has been broadly reported on the basis of the bulk tissue analysis of the cytokine release and the scRNA-seq analysis (*31*, *36*); however, these studies do not provide a clear picture of the interaction between macrophages and fibroblasts and remodeled ECMs. As a result, the fibrosis formation process at the tissue and cellular level is still unclear. This has led to difficulties identifying cellular and molecular targets for anti-fibrosis therapies. In the current study, a macrophage and fibroblast cocultured fibrosis tissue model was developed to allow the investigation of immune-stroma interactions in a fibrosis microenvironment. Using tissue microfabrication method, we created micropillar-based lung microtissues with controlled topographies to mimic the alignment of fibroblasts and collagen fibers in the fibrotic lung tissue. We showed that profibrotic macrophages were able to sense and adapt to the topographic cues and become elongated and align along the fibroblasts and collagen fibers. At the single-cell level, such co-alignment allowed ample contact between the macrophage and the fibroblast. Because it has been shown that proximity is crucial for the cross-talk between macrophages and fibroblasts (*15*), the increased level of physical contacts between these two cell types likely enhanced the cellular signal transduction and latent TGF-β activation and promoted macrophage-mediated myofibroblast differentiation. At the tissue level, the report on the distribution of profibrotic macrophages in lung fibrosis and their relative position to the fibroblasts is limited. Through histological analysis, we showed that CD206^+^ profibrotic macrophages were widely distributed in the fibrotic lung tissue and embedded in the highly deformed ECM along with α-SMA–positive myofibroblasts. The coculture model in the current study recapitulated this effect and demonstrated that extensive co-alignment between the macrophages and fibroblasts enabled the widespread fibrosis response in the tissue, as seen in lung fibrosis.

Lung fibrosis is associated with a mechanically active tissue microenvironment. By modeling the fibrotic tissue, the current study provides an excellent opportunity to examine the macrophage mechanobiology in a microenvironment abundant in mechanical signals. Macrophages are known to be mechanically sensitive and M2 macrophages have been shown to align on the microgrooves created on 2D elastic substrate (*20*). Increased alignment promoted the M2 differentiation of the macrophages. Aligned patterns with feature sizes in the micron level were shown to promote M2 macrophage differentiation through forced alignment, whereas irregular topographies with similar dimensions produce circularly spreading macrophages with M1 characteristics (*20*, *37*–*42*). In the current study, we showed that fibroblast-mediated cell and ECM fiber alignment provided an instructive microenvironment for the macrophage mechanical activation, which may imply a mechanism of macrophage recruitment and activation in fibrotic diseases. We further showed that mechanical activation of macrophages led to elevated level of active TGF-β secretion and myofibroblast differentiation, suggesting that mechanical activation is an important step in the activation of the pro-fibrotic functions of macrophages. In contrast, IL-3/IL-14–polarized M2a macrophages seeded in pure collagen matrix were not able to generate sufficient contractile force to compact the collagen matrix and form a microtissue (fig. S5B), and non-polarized monocytes, when introduced to the fibroblast-populated collagen microtissue, were not able to induce α-SMA expression in the fibroblasts (fig. S4). Together, these experiments show that both the IL-4/13 cytokine condition and the mechanobiological cue are needed for the polarization and mechanical activation of the pro-fibrotic macrophages.

In addition to surface topographies, human and mouse primary macrophages were shown to respond to the elastic modulus (“stiffness”) of their substrates (*43*). In general, macrophages attaching to soft materials exhibit reduced activation into profibrotic M2 macrophages compared to those grown on stiffer substrates (*44*–*48*). Furthermore, macrophages are able to sense and follow changes in the deformation of collagen networks caused by the contracting fibroblasts over millimeter distances (*49*). To test the effect of substrate stiffness on the macrophage mechanical activation, we performed macrophage adhesion assay on both physiologically soft PDMS surface and hard plastic surface with and without PFD treatment. Although PFD was found to have a dose dependent inhibitory effect on the monocyte to M2 macrophage differentiation, it did not significantly reduce the number of adhered monocyte/macrophages seeded on the plastic dish (fig. S10), likely due to the difficulty to disrupt the preestablished strong macrophage adhesion on the stiff substrate. In contrast, PFD pretreatment significantly reduced the M2 macrophage adhesion on physiologically soft PDMS surface (1 kPa) (fig. S11). This is likely due to the weak adhesion formed between macrophages and the soft substrate. These results have direct implication for PFD-induced macrophage loss observed in our cocultured microtissue models. In our prior work (*50*–*53*), we documented that fibroblast-populated microtissues exhibit a stiffness of 5 kPa, aligning with the stiffness of physiologically soft PDMS. This low substrate stiffness likely leads to weak macrophage adhesion to the microtissue, which PFD treatment easily disrupts.

Current therapies for fibrotic diseases are very limited. PFD and Nintedanib are the only two drugs approved by FDA to treat IPF, but they can only slow down the disease progression and cannot stop or reserve the disease (*32*). The mechanisms of action of PFD and nintedanib are not fully understood. PFD is generally believed to act on fibroblasts through inhibiting TGF-β pathway, but recent studies have shown that PFD can also modulate the macrophage polarization and profibrotic activities (*29*, *30*). In the current study, we showed that PFD strongly affected the M2 macrophage adhesion on the fibroblast-populated stromal tissue through inhibiting the ROCK2 and integrin αMβ2. Because macrophage adhesion is critical to their recruitment to the injury site, this finding suggests a previously unknown mechanism of action of PFD on macrophage-mediated fibrosis. In a recent study, a pan-ROCK inhibitor (WXWH0265) suppressed M2 macrophage polarization through inhibition of ROCK activity via down-regulated signal transducer and activator of transcription 3 phosphorylation in the treatment of pulmonary fibrosis (*54*). The same cellular and molecular target and disease type in this existing study suggests that this signaling pathway may play an important role in our observed results of ROCK2 reduction. Further histological and single-cell studies on PFD-treated donor lung samples are needed to determine the clinical relevancy of this finding. If confirmed, then this may open an avenue for the development of anti-fibrosis drugs that target immune cells. The above findings have been enabled by the cocultured, force-sensing microtissue model developed in this study. Compared to the previous cocultured models of immune cells and stromal cells (*15*, *55*–*58*), the current model has unique features to allow the manipulation of the mechanical microenvironment and the measurement of the change in the tissue mechanical properties. As a result, it provides perspectives to the immune-stroma interaction and can unveil previously unknown disease and therapeutic mechanisms. Together, this novel system has the potential to become a powerful tool to study the complex immune–stromal cell interactions and the mechanism of action of anti-fibrosis drugs.

## MATERIALS AND METHODS

### Peripheral blood monocyte isolation and pro-fibrotic macrophage differentiation

The whole blood was collected at the Clinical and Translational Research Center in Buffalo according to the approved protocols with the University at Buffalo Research Subjects Institutional Review Board and with informed consent obtained from all donors. The peripheral blood mononuclear cells were isolated through density gradient centrifugation, where 18 ml of blood was mixed with Hanks’ balanced salt solution (HBSS; Gibco, 14175095) before being poured onto Ficoll-Paque Plus (GE Healthcare, GE17-1440-03). The layers were separated through centrifugation, and the cells from the interphase were washed twice with HBSS before being resuspended in 5 ml of HBSS for monocyte purification. The classical monocytes were isolated using biotin-conjugated antibodies and streptavidin-conjugated magnetic beads to deplete labeled cells and were then conditioned into resting macrophages (M0) using RPMI 1640 supplemented with 10% fetal bovine serum (FBS; R&D Systems, S11150H) and 1% penicillin/streptomycin (P/S; Gibco, 15140122) for 7 days. After 4 days, fresh medium with M-CSF (50 ng/ml; Gibco, PHC9504) was added, and mature macrophages were polarized by adding IL-4 (20 ng/ml; A42602, Invitrogen) and IL-13 (20 ng/ml; Invitrogen, A42526) to generate M2 macrophages for an additional 24 hours. Last, for monocytes or macrophages cultured on glass coverslip, the culture surface of glass coverslips was coated with rat tail collagen type I (Advanced BioMatrix, 5153) before cell seeding.

### NHLF cell culture and coculture assays

Primary NHLFs were purchased from Lonza and subcultured for up to six passages in manufacturer supplied growth medium (FGM-2 BulletKit; Lonza, CC3132). The microtissues populated with NHLF were grown for 2 days before the addition of M2 macrophages at a ratio of 4:1 (M2:NHLF). Cocultures of macrophages and NHLF were carried out for 3 days in macrophage base medium containing RPMI 1640, 10% FBS, and 1% P/S.

### Micropillar device fabrication and microtissue seeding

The fabrication of micropillar arrays involved the utilization of a multilayer microlithography technique, as previously described (*50*). In summary, the process included the following steps: First, SU-8 masters were created by spin coating multiple layers of SU-8 photoresists, followed by alignment, exposure, and baking. To achieve a cross-sectional difference between the micropillar head and leg sections, a thin layer of SU-8 doped with S1813 was deposited, acting as a barrier to prevent ultraviolet (UV) light penetration into the leg section. UV exposure was conducted using an OAI mask aligner equipped with a U-360 band pass filter. Subsequently, PDMS (Sylgard 184, Dow Corning) stamps were cast over the SU-8 master using a 10:1 mixing ratio. The micropillar devices were then generated through replica molding from the PDMS stamps, using P35 petri dishes as the mold. For microtissue seeding, a previously established protocol was used (*50*). The micropillar devices were sterilized and treated with Pluronic F-127 (P2443, Sigma-Aldrich) to prevent nonspecific cell adhesion to the PDMS surface. NHLFs were mixed with rat tail collagen type I (Advanced BioMatrix, 5153) at a final concentration of 2 mg/ml. The mixture was introduced into the microwells by centrifugation. Subsequently, the collagen solution was crosslinked and maintained in appropriate growth media within a CO_2_ incubator. After the microtissue formation, additional NHLF cells were introduced to the surface of the microtissues before loading the macrophages.

For tracing purposes in some conditions, cells were fluorescently labelled. One million human macrophages and NHLF were suspended in 1 ml of serum-free RPMI 1640 medium. To label the cells, 5 μl of Vybrant DiO Cell-Labeling Solution (Invitrogen, V22886) was added to the NHLF suspension, while 5 μl of Vybrant DiI Cell-Labeling Solution (Invitrogen, V22885) was added to the macrophage suspension. The cells were then incubated at 37°C, 95% humidity, and 5% CO_2_ for 10 min before being washed three times with phosphate-buffered saline (PBS; Gibco, 10010023).

### Pharmacological treatment

Anti-fibrotic drugs PFD (TCI America, P1871) and KD025 (MedChemExpress, HY-15307) were purchased from commercial suppliers. To induce myofibroblast differentiation of NHLFs in a macrophage-free culture system, 5 ng/ml of TGF-ß1 (Sigma Aldrich, T5050) was added to the macrophage base medium and maintained for 3 days. To test the anti-fibrosis drugs in a therapeutic manner, human monocytes derived M0 macrophages were cultured in macrophage base medium supplemented with M-CSF, IL-4, and IL-13 to promote M2 polarization. After 24 hours, macrophages were collected and loaded onto NHLF populated microtissues, which were maintained in macrophage base medium for 3 days to induce fibrosis. On day 3, the culture medium was refreshed with PFD (0, 10, or 1000 μg/ml) coadministered into the coculture system, which was maintained for another 3 days.

To test the anti-fibrosis drugs in a preventative manner, human monocytes derived M0 macrophages were cultured in macrophage base medium supplemented with M-CSF, IL-4, and IL-13 to promote M2 polarization. After 24 hours, macrophages were collected and loaded onto NHLF populated microtissues. PFD was coadministered at the same time at 0, 10, or 1000 μg/ml into the cocultured system, which was maintained for 3 days in macrophage base medium. To investigate the anti-fibrotic effect of PFD on macrophages, human monocytes derived M0 macrophages were cultured in macrophage base medium supplemented with PFD for 24 hours before treatment with cytokines. After another 24 hours, M0 macrophages were stimulated with M-CSF and IL-4 and IL-13 to promote M2 polarization. Then, macrophages were collected and loaded onto normal NHLF populated microtissues, which were maintained in macrophage base medium for 3 days.

### Microtissue contraction force measurement

The force of spontaneous contraction generated by individual microtissues was determined by measuring the deflection of micropillars using cantilever bending theory. The deflection of the micropillars was measured by comparing the deflected position of the centroid of each pillar top to the centroid of its base using phase contrast microscopy. The microtissue contraction force was measured for the same batch of samples over different time points for each pharmacological condition. Image analysis was conducted using ImageJ software, and the absolute values of the calculated force from each micropillar were added together and divided by 2 to obtain the collective force generated by one microtissue.

### Active TGF-β measurements

To quantify the amount of active TGF-β, transformed mink lung epithelial cells (TMLC) reporters were used. These cells produce luciferase in response to TGF-β under the control of the plasminogen activator inhibitor-1(PAI-1) promoter. To determine the amount of TGF-β in culture supernatants, TMLCs (40,000 cells/cm^2^) were allowed to adhere for 3 hours, after which they were exposed to conditioned media or native (active TGF-β) for 24 hours. TMLCs were then lysed, and the luminescence was measured using a luciferase assay kit (Promega) and luminometer (Centro LB, Berthold Technologies). All results were adjusted to account for the baseline luciferase production of TMLCs in the absence of TGF-β. TGF-β concentrations were determined using standard curves generated with known concentrations of active TGF-β in serum-free medium (ranging from 0 to 8000 pg/ml) (fig. S9).

### Immunofluorescence and image analysis

The human lung cell–populated microtissues were fixed with 3.7% formaldehyde. Then, cold methanol was used to permeabilize the cells, allowing the antibodies to penetrate and bind to their target proteins. The cells were then blocked with 3% bovine serum albumin to prevent nonspecific binding of the antibodies. The primary antibodies used were against α-SMA (Abcam, ab7817; 1:300), mannose receptor (CD206) (Abcam, ab64693; 1:100), TGF-β1 (Abcam, ab215715; 1:200), fibronectin (Invitrogen, PA5-29578; 1:200), integrin αM (CD11b) (Invitrogen, 53-0112-82; 1:100), integrin β2 (CD18) (Abcam, ab307406; 1:100), and Rho-associated coiled-coil containing protein kinase 2 (ROCK2) (Santa Cruz Biotechnology, sc-398519; 1:100). These antibodies were labeled with fluorophore-conjugated, anti–immunoglobulin G antibodies (Thermo Fisher Scientific, Alexa Fluor, 1:400) to enable their detection. The nuclei were counterstained with Hoechst 33342 (Thermo Fisher Scientific, 1:1000).

To measure the fluorescence intensity of α-SMA in different microtissues under varied pharmacological conditions, images were taken on a Nikon Ti-U microscope equipped with a Hamamatsu ORCA-ER CCD camera using a 10× Plan Fluor objective under identical imaging conditions. Fluorescence intensity was measured in ImageJ by taking the integrated intensity of a region of interest bounded by the inner edge of micropillars and then subtracting the background intensity. Confocal images of the microtissues were taken on a Leica Stellaris 5 fluorescence microscope with 10× air objective and 40× oil immersion objective. An optical slice of 1 μm was used for all channels, and the stack of images was processed using the *Z* stack tool in Leica LAS X software package.

The number of macrophages remaining attached on the surface of microtissues and glass coverslips was quantified from single-color fluorescence images using ImageJ, a software program developed by the US National Institutes of Health, Bethesda, MA, USA, and available for free download (http://imagej.nih.gov/ij/; 1997–2013).

### Histologic analysis of lung sections

Human lung tissue samples from patients diagnosed with IPF were obtained from the National Disease Research Interchange. The tissue was cut into small pieces measuring 0.3 to 0.5 cm in one dimension and embedded in Tissue-Tek O.C.T. Compound (Sakura Finetek, 4583). The embedded tissue was rapidly frozen on dry ice and cut into 15-μm cryostat sections. The sections were then mounted on microscope slides (MS-CS-90, 45o Corners, Stellar Scientific) and subjected to immunofluorescent staining and H&E staining.

### Scanning electron microscopy

The microdevices containing NHLFs with or without macrophages were fixed using a solution of 2% glutaraldehyde (Acros Organics) in PBS (containing Ca^2+^/Mg^2+^) for 1 hour at 4°C. Next, the samples were dehydrated with ethanol in increments of 10% from 50 to 100% for 10 min each, followed by critical point drying for 1 hour using hexamethyldisilazane (Alfa Aesar) in a chemical hood. The scanning electron microscopy (SEM) was performed using the Hitachi SU70 Field Emission Scanning Electron Microscope.

### Single-cell RNA-seq data mining

To compile a list of genes associated with the activation of mechanobiological pathways in macrophages, a thorough search was conducted in the National Library of Medicine’s database for *Homo sapiens*. This comprehensive search yielded a carefully curated list of 224 genes, which was subsequently cross-referenced with NCBI GEO datasets GSE122960 and GSE228232. The scRNA-seq data from GSE122960 were visualized using UCSC CellBrowser, a tool developed by Maximilian Haeussler (www.nupulmonary.org/resources/). In addition, the processed scRNA-seq data from GSE228232 were visualized using Single Cell Portal (https://singlecell.broadinstitute.org/single_cell). The GO enrichment data were collected from the openly accessible dataset GSE122960 in the NCBI GEO database.

For the generation of *t*-distributed stochastic neighbor embedding (*t*-SNE) plots for alveolar macrophages (Fig. 1, C and D), cells identified as macrophages through individual annotation of single-cell RNA-seq data from eight normal lungs and eight fibrotic lungs were aggregated. On the *t*-SNE plot representing all 16 samples, cells were categorized on the basis of their origin, distinguishing between those from donors and those from patients with pulmonary fibrosis. The color scheme was used to differentiate between these two groups.

### Statistical analysis

All experiments were conducted with a minimum of three biological replicates. The quantitative data are presented as means ± SD.Statistical differences between groups were evaluated using analysis of variance (ANOVA) followed by a post hoc Tukey’s multiple comparisons test and Dunnett’s multiple comparisons test. The significance levels were set at *P ≤ 0.05, **P ≤ 0.01, and ***P ≤ 0.001. For experiments comparing two groups, a two-tailed paired Student’s *t* test was performed. Statistically significant differences were denoted as #*P* ≤ 0.05, ##*P* ≤ 0.01, and ###*P* ≤ 0.001.

## References

[1] F. J. Martinez, H. R. Collard, A. Pardo, G. Raghu, L. Richeldi, M. Selman, J. J. Swigris, H. Taniguchi, A. U. Wells, Idiopathic pulmonary fibrosis. Nat. Rev. Dis. Primers. 3, 17074 (2017). 29052582 10.1038/nrdp.2017.74

[2] M. Ochs, S. Timm, S. Elezkurtaj, D. Horst, J. Meinhardt, F. L. Heppner, S. Weber-Carstens, A. C. Hocke, M. Witzenrath, Collapse induration of alveoli is an ultrastructural finding in a COVID-19 patient. Eur. Respir. J. 57, 2004165 (2021). 33446606 10.1183/13993003.04165-2020 PMC7815985

[3] J. Snijder, J. Peraza, M. Padilla, K. Capaccione, M. M. Salvatore, Pulmonary fibrosis: A disease of alveolar collapse and collagen deposition. Expert Rev. Respir. Med. 13, 615–619 (2019). 31117843 10.1080/17476348.2019.1623028

[4] M. S. Wilson, T. A. Wynn, Pulmonary fibrosis: Pathogenesis, etiology and regulation. Mucosal Immunol. 2, 103–121 (2009). 19129758 10.1038/mi.2008.85 PMC2675823

[5] T. Fabre, A. M. S. Barron, S. M. Christensen, S. Asano, K. Bound, M. P. Lech, M. H. Wadsworth II, X. Chen, C. Wang, J. Wang, J. McMahon, F. Schlerman, A. White, K. M. Kravarik, A. J. Fisher, L. A. Borthwick, K. M. Hart, N. C. Henderson, T. A. Wynn, K. Dower, Identification of a broadly fibrogenic macrophage subset induced by type 3 inflammation. Sci Immunol 8, eadd8945 (2023). 37027478 10.1126/sciimmunol.add8945

[6] A. J. Byrne, T. M. Maher, C. M. Lloyd, Pulmonary macrophages: A new therapeutic pathway in fibrosing lung disease? Trends Mol. Med. 22, 303–316 (2016). 26979628 10.1016/j.molmed.2016.02.004

[7] J. S. Duffield, M. Lupher, V. J. Thannickal, T. A. Wynn, Host responses in tissue repair and fibrosis. Annu. Rev. Pathol. 8, 241–276 (2013). 23092186 10.1146/annurev-pathol-020712-163930 PMC3789589

[8] S. A. Eming, T. A. Wynn, P. Martin, Inflammation and metabolism in tissue repair and regeneration. Science 356, 1026–1030 (2017). 28596335 10.1126/science.aam7928

[9] R. Mirza, L. A. DiPietro, T. J. Koh, Selective and specific macrophage ablation is detrimental to wound healing in mice. Am. J. Pathol. 175, 2454–2462 (2009). 19850888 10.2353/ajpath.2009.090248 PMC2789630

[10] A. S. MacLeod, J. N. Mansbridge, The innate immune system in acute and chronic wounds. Adv. Wound Care (New Rochelle) 5, 65–78 (2016). 26862464 10.1089/wound.2014.0608 PMC4742992

[11] J. S. Duffield, S. J. Forbes, C. M. Constandinou, S. Clay, M. Partolina, S. Vuthoori, S. Wu, R. Lang, J. P. Iredale, Selective depletion of macrophages reveals distinct, opposing roles during liver injury and repair. J. Clin. Invest. 115, 56–65 (2005). 15630444 10.1172/JCI22675 PMC539199

[12] J. M. Fritz, M. A. Tennis, D. J. Orlicky, H. Lin, C. Ju, E. F. Redente, K. S. Choo, T. A. Staab, R. J. Bouchard, D. T. Merrick, A. M. Malkinson, L. D. Dwyer-Nield, Depletion of tumor-associated macrophages slows the growth of chemically induced mouse lung adenocarcinomas. Front. Immunol. 5, 587 (2014). 25505466 10.3389/fimmu.2014.00587 PMC4243558

[13] Z. Zhu, J. Ding, Z. Ma, T. Iwashina, E. E. Tredget, Systemic depletion of macrophages in the subacute phase of wound healing reduces hypertrophic scar formation. Wound Repair Regen. 24, 644–656 (2016). 27169512 10.1111/wrr.12442

[14] A. V. Misharin, L. Morales-Nebreda, P. A. Reyfman, C. M. Cuda, J. M. Walter, A. C. McQuattie-Pimentel, C.-I. Chen, K. R. Anekalla, N. Joshi, K. J. N. Williams, H. Abdala-Valencia, T. J. Yacoub, M. Chi, S. Chiu, F. J. Gonzalez-Gonzalez, K. Gates, A. P. Lam, T. T. Nicholson, P. J. Homan, S. Soberanes, S. Dominguez, V. K. Morgan, R. Saber, A. Shaffer, M. Hinchcliff, S. A. Marshall, A. Bharat, S. Berdnikovs, S. M. Bhorade, E. T. Bartom, R. I. Morimoto, W. E. Balch, J. I. Sznajder, N. S. Chandel, G. M. Mutlu, M. Jain, C. J. Gottardi, B. D. Singer, K. M. Ridge, N. Bagheri, A. Shilatifard, G. R. S. Budinger, H. Perlman, Monocyte-derived alveolar macrophages drive lung fibrosis and persist in the lung over the life span. J. Experim. Med. 214, 2387–2404 (2017). 10.1084/jem.20162152 PMC5551573 28694385

[15] M. Lodyga, E. Cambridge, H. M. Karvonen, P. Pakshir, B. Wu, S. Boo, M. Kiebalo, R. Kaarteenaho, M. Glogauer, M. Kapoor, K. Ask, B. Hinz, Cadherin-11–mediated adhesion of macrophages to myofibroblasts establishes a profibrotic niche of active TGF-β. Sci. Signal. 12, eaao3469 (2019). 30647145 10.1126/scisignal.aao3469

[16] P. Pakshir, N. Noskovicova, M. Lodyga, D. O. Son, R. Schuster, A. Goodwin, H. Karvonen, B. Hinz, The myofibroblast at a glance. J. Cell Sci. 133, jcs227900 (2020). 32651236 10.1242/jcs.227900

[17] A. J. Haak, Q. Tan, D. J. Tschumperlin, Matrix biomechanics and dynamics in pulmonary fibrosis. Matrix Biol. 73, 64–76 (2018). 29274939 10.1016/j.matbio.2017.12.004 PMC6013326

[18] S. Adams, L. M. Wuescher, R. Worth, E. Yildirim-Ayan, Mechano-immunomodulation: Mechanoresponsive changes in macrophage activity and polarization. Ann. Biomed. Eng. 47, 2213–2231 (2019). 31218484 10.1007/s10439-019-02302-4 PMC7043232

[19] N. Jain, J. Moeller, V. Vogel, Mechanobiology of macrophages: How physical factors coregulate macrophage plasticity and phagocytosis. Annu. Rev. Biomed. Eng. 21, 267–297 (2019). 31167103 10.1146/annurev-bioeng-062117-121224

[20] F. Y. McWhorter, T. Wang, P. Nguyen, T. Chung, W. F. Liu, Modulation of macrophage phenotype by cell shape. Proc. Natl. Acad. Sci. U.S.A. 110, 17253–17258 (2013). 24101477 10.1073/pnas.1308887110 PMC3808615

[21] P. J. Murray, J. E. Allen, S. K. Biswas, E. A. Fisher, D. W. Gilroy, S. Goerdt, S. Gordon, J. A. Hamilton, L. B. Ivashkiv, T. Lawrence, M. Locati, A. Mantovani, F. O. Martinez, J. L. Mege, D. M. Mosser, G. Natoli, J. P. Saeij, J. L. Schultze, K. A. Shirey, A. Sica, J. Suttles, I. Udalova, J. A. van Ginderachter, S. N. Vogel, T. A. Wynn, Macrophage activation and polarization: Nomenclature and experimental guidelines. Immunity 41, 14–20 (2014). 25035950 10.1016/j.immuni.2014.06.008 PMC4123412

[22] S. Gordon, A. Pluddemann, Tissue macrophages: Heterogeneity and functions. BMC Biol. 15, 53 (2017). 28662662 10.1186/s12915-017-0392-4 PMC5492929

[23] M. L. Novak, T. J. Koh, Macrophage phenotypes during tissue repair. J. Leukoc. Biol. 93, 875–881 (2013). 23505314 10.1189/jlb.1012512 PMC3656331

[24] P. Ramachandran, A. Pellicoro, M. A. Vernon, L. Boulter, R. L. Aucott, A. Ali, S. N. Hartland, V. K. Snowdon, A. Cappon, T. T. Gordon-Walker, M. J. Williams, D. R. Dunbar, J. R. Manning, N. van Rooijen, J. A. Fallowfield, S. J. Forbes, J. P. Iredale, Differential Ly-6C expression identifies the recruited macrophage phenotype, which orchestrates the regression of murine liver fibrosis. Proc. Natl. Acad. Sci. U.S.A. 109, E3186–E3195 (2012). 23100531 10.1073/pnas.1119964109 PMC3503234

[25] E. A. Ayaub, A. Dubey, J. Imani, F. Botelho, M. R. J. Kolb, C. D. Richards, K. Ask, Overexpression of OSM and IL-6 impacts the polarization of pro-fibrotic macrophages and the development of bleomycin-induced lung fibrosis. Sci. Rep. 7, 13281 (2017). 29038604 10.1038/s41598-017-13511-z PMC5643520

[26] N. C. Henderson, F. Rieder, T. A. Wynn, Fibrosis: From mechanisms to medicines. Nature 587, 555–566 (2020). 33239795 10.1038/s41586-020-2938-9 PMC8034822

[27] P. W. Noble, C. Albera, W. Z. Bradford, U. Costabel, R. M. Du Bois, E. A. Fagan, R. S. Fishman, I. Glaspole, M. K. Glassberg, L. Lancaster, D. J. Lederer, J. A. Leff, S. D. Nathan, C. A. Pereira, J. J. Swigris, D. Valeyre, T. E. King, Pirfenidone for idiopathic pulmonary fibrosis: Analysis of pooled data from three multinational phase 3 trials. Eur. Respir. J. 47, 243–253 (2016). 26647432 10.1183/13993003.00026-2015 PMC4697914

[28] S. H. Hamidi, S. Kadamboor Veethil, S. H. Hamidi, Role of pirfenidone in TGF-β pathways and other inflammatory pathways in acute respiratory syndrome coronavirus 2 (SARS-CoV-2) infection: A theoretical perspective. Pharmacol. Rep. 73, 712–727 (2021). 33880743 10.1007/s43440-021-00255-x PMC8057922

[29] H. Ying, M. Fang, Q. Q. Hang, Y. Chen, X. Qian, M. Chen, Pirfenidone modulates macrophage polarization and ameliorates radiation-induced lung fibrosis by inhibiting the TGF-β1/Smad3 pathway. J. Cell. Mol. Med. 25, 8662–8675 (2021). 34327818 10.1111/jcmm.16821 PMC8435416

[30] M. Toda, S. Mizuguchi, Y. Minamiyama, H. Yamamoto-Oka, T. Aota, S. Kubo, N. Nishiyama, T. Shibata, S. Takemura, Pirfenidone suppresses polarization to M2 phenotype macrophages and the fibrogenic activity of rat lung fibroblasts. J. Clin. Biochem. Nutr. 63, 58–65 (2018). 30087545 10.3164/jcbn.17-111 PMC6064814

[31] P. A. Reyfman, J. M. Walter, N. Joshi, K. R. Anekalla, A. C. McQuattie-Pimentel, S. Chiu, R. Fernandez, M. Akbarpour, C. I. Chen, Z. Ren, R. Verma, H. Abdala-Valencia, K. Nam, M. Chi, S. Han, F. J. Gonzalez-Gonzalez, S. Soberanes, S. Watanabe, K. J. N. Williams, A. S. Flozak, T. T. Nicholson, V. K. Morgan, D. R. Winter, M. Hinchcliff, C. L. Hrusch, R. D. Guzy, C. A. Bonham, A. I. Sperling, R. Bag, R. B. Hamanaka, G. M. Mutlu, A. V. Yeldandi, S. A. Marshall, A. Shilatifard, L. A. N. Amaral, H. Perlman, J. I. Sznajder, A. C. Argento, C. T. Gillespie, J. Dematte, M. Jain, B. D. Singer, K. M. Ridge, A. P. Lam, A. Bharat, S. M. Bhorade, C. J. Gottardi, G. R. S. Budinger, A. V. Misharin, Single-cell transcriptomic analysis of human lung provides insights into the pathobiology of pulmonary fibrosis. Am. J. Respir. Crit. Care Med. 199, 1517–1536 (2019). 30554520 10.1164/rccm.201712-2410OC PMC6580683

[32] K. M. Roach, E. Castells, K. Dixon, S. Mason, G. Elliott, H. Marshall, M. A. Poblocka, S. Macip, M. Richardson, L. Khalfaoui, P. Bradding, Evaluation of pirfenidone and nintedanib in a human lung model of fibrogenesis. Front. Pharmacol. 12, 679388 (2021). 34712131 10.3389/fphar.2021.679388 PMC8546112

[33] L. Richeldi, S. Fletcher, H. Adamali, N. Chaudhuri, S. Wiebe, S. Wind, K. Hohl, A. Baker, R. Schlenker-Herceg, S. Stowasser, T. M. Maher, No relevant pharmacokinetic drug–drug interaction between nintedanib and pirfenidone. Eur. Respir. J. 53, 1801060 (2019). 30442716 10.1183/13993003.01060-2018

[34] V. S. Meli, P. K. Veerasubramanian, H. Atcha, Z. Reitz, T. L. Downing, W. F. Liu, Biophysical regulation of macrophages in health and disease. J. Leukoc. Biol. 106, 283–299 (2019). 30861205 10.1002/JLB.MR0318-126R PMC7001617

[35] M. Jagasia, A. Lazaryan, C. R. Bachier, A. Salhotra, D. J. Weisdorf, B. Zoghi, J. Essell, L. Green, O. Schueller, J. Patel, A. Zanin-Zhorov, J. M. Weiss, Z. Yang, D. Eiznhamer, S. K. Aggarwal, B. R. Blazar, S. J. Lee, ROCK2 inhibition with belumosudil (KD025) for the treatment of chronic graft-versus-host disease. J. Clin. Oncol. 39, 1888–1898 (2021). 33877856 10.1200/JCO.20.02754 PMC8189612

[36] E. Ayaub, S. Poli, J. Ng, T. Adams, J. Schupp, L. Quesada-Arias, F. Poli, C. Cosme, M. Robertson, J. Martinez-Manzano, X. Liang, J. Villalba, J. Lederer, S. Chu, B. Raby, G. Washko, C. Coarfa, M. Perrella, S. El-Chemaly, N. Kaminski, I. Rosas, Single cell RNA-seq and mass cytometry reveals a novel and a targetable population of macrophages in idiopathic pulmonary fibrosis. bioRxiv [Preprint]. 2021. 10.1101/2021.01.04.425268.

[37] T. Wang, T. U. Luu, A. Chen, M. Khine, W. F. Liu, Topographical modulation of macrophage phenotype by shrink-film multi-scale wrinkles. Biomater. Sci. 4, 948–952 (2016). 27125253 10.1039/c6bm00224b PMC9061279

[38] T. U. Luu, S. C. Gott, B. W. Woo, M. P. Rao, W. F. Liu, Micro- and nanopatterned topographical cues for regulating macrophage cell shape and phenotype. ACS Appl. Mater. Interfaces 7, 28665–28672 (2015). 26605491 10.1021/acsami.5b10589 PMC4797644

[39] S. Chen, J. A. Jones, Y. Xu, H. Y. Low, J. M. Anderson, K. W. Leong, Characterization of topographical effects on macrophage behavior in a foreign body response model. Biomaterials 31, 3479–3491 (2010). 20138663 10.1016/j.biomaterials.2010.01.074 PMC2837101

[40] H. Moon, C. V. Cremmel, A. Kulpa, N. A. Jaeger, R. Kappelhoff, C. M. Overall, J. D. Waterfield, D. M. Brunette, Novel grooved substrata stimulate macrophage fusion, CCL2 and MMP-9 secretion. J. Biomed. Mater. Res. A 104, 2243–2254 (2016). 27102570 10.1002/jbm.a.35757

[41] Y. Zhang, X. Cheng, J. A. Jansen, F. Yang, J. van den Beucken, Titanium surfaces characteristics modulate macrophage polarization. Mater. Sci. Eng. C Mater. Biol. Appl. 95, 143–151 (2019). 30573235 10.1016/j.msec.2018.10.065

[42] C. E. Witherel, K. Sao, B. K. Brisson, B. Han, S. W. Volk, R. J. Petrie, L. Han, K. L. Spiller, Regulation of extracellular matrix assembly and structure by hybrid M1/M2 macrophages. Biomaterials 269, 120667 (2021). 33450585 10.1016/j.biomaterials.2021.120667 PMC7870567

[43] P. A. Janmey, B. Hinz, C. A. McCulloch, Physics and physiology of cell spreading in two and three dimensions. Physiology (Bethesda) 36, 382–391 (2021). 34704856 10.1152/physiol.00020.2021 PMC8560373

[44] E. F. Irwin, K. Saha, M. Rosenbluth, L. J. Gamble, D. G. Castner, K. E. Healy, Modulus-dependent macrophage adhesion and behavior. J. Biomater. Sci. Polym. Ed. 19, 1363–1382 (2008). 18854128 10.1163/156856208786052407

[45] K. M. Adlerz, H. Aranda-Espinoza, H. N. Hayenga, Substrate elasticity regulates the behavior of human monocyte-derived macrophages. Eur. Biophys. J. 45, 301–309 (2016). 26613613 10.1007/s00249-015-1096-8

[46] A. K. Blakney, M. D. Swartzlander, S. J. Bryant, The effects of substrate stiffness on the in vitro activation of macrophages and in vivo host response to poly(ethylene glycol)-based hydrogels. J. Biomed. Mater. Res. A 100, 1375–1386 (2012). 22407522 10.1002/jbm.a.34104 PMC3339197

[47] S. Fereol, R. Fodil, B. Labat, S. Galiacy, V. M. Laurent, B. Louis, D. Isabey, E. Planus, Sensitivity of alveolar macrophages to substrate mechanical and adhesive properties. Cell Motil. Cytoskeleton 63, 321–340 (2006). 16634082 10.1002/cm.20130

[48] R. A. Scott, K. L. Kiick, R. E. Akins, Substrate stiffness directs the phenotype and polarization state of cord blood derived macrophages. Acta Biomater. 122, 220–235 (2021). 33359292 10.1016/j.actbio.2020.12.040 PMC7904389

[49] P. Pakshir, M. Alizadehgiashi, B. Wong, N. M. Coelho, X. Chen, Z. Gong, V. B. Shenoy, C. A. McCulloch, B. Hinz, Dynamic fibroblast contractions attract remote macrophages in fibrillar collagen matrix. Nat. Commun. 10, 1850 (2019). 31015429 10.1038/s41467-019-09709-6 PMC6478854

[50] M. Asmani, S. Velumani, Y. Li, N. Wawrzyniak, I. Hsia, Z. Chen, B. Hinz, R. Zhao, Fibrotic microtissue array to predict anti-fibrosis drug efficacy. Nat. Commun. 9, 2066 (2018). 29802256 10.1038/s41467-018-04336-z PMC5970268

[51] Z. Chen, J. Lu, C. Zhang, I. Hsia, X. Yu, L. Marecki, E. Marecki, M. Asmani, S. Jain, S. Neelamegham, R. Zhao, Microclot array elastometry for integrated measurement of thrombus formation and clot biomechanics under fluid shear. Nat. Commun. 10, 2051 (2019). 31053712 10.1038/s41467-019-10067-6 PMC6499828

[52] M. Asmani, C. Kotei, I. Hsia, L. Marecki, T. Wang, C. Zhou, R. Zhao, Cyclic stretching of fibrotic microtissue array for evaluation of anti-fibrosis drugs. Cellul. Molec. Bioeng. 12, 529–540 (2019). 10.1007/s12195-019-00590-3 PMC6816662 31719931

[53] I. Hsia, M. Asmani, R. Zhao, Predicting the preclinical efficacy of anti-fibrosis agents using a force-sensing fibrosis on chip system. Biosens. Bioelectron. 228, 115194 (2023). 36933322 10.1016/j.bios.2023.115194

[54] Q. Li, Y. Cheng, Z. Zhang, Z. Bi, X. Ma, Y. Wei, X. Wei, Inhibition of ROCK ameliorates pulmonary fibrosis by suppressing M2 macrophage polarisation through phosphorylation of STAT3. Clin. Transl. Med. 12, e1036 (2022). 36178087 10.1002/ctm2.1036 PMC9523675

[55] F. Ullm, P. Riedl, A. Machado De Amorim, A. Patzschke, R. Weiß, S. Hauschildt, K. Franke, U. Anderegg, T. Pompe, 3D scaffold-based macrophage fibroblast coculture model reveals IL-10 dependence of wound resolution phase. Adv. Biosyst. 4, e1900220 (2020). 32293120 10.1002/adbi.201900220

[56] Y. Tan, A. Suarez, M. Garza, A. A. Khan, J. Elisseeff, D. Coon, Human fibroblast-macrophage tissue spheroids demonstrate ratio-dependent fibrotic activity for in vitro fibrogenesis model development. Biomater. Sci. 8, 1951–1960 (2020). 32057054 10.1039/c9bm00900k PMC7179997

[57] P. Vijayaraj, A. Minasyan, A. Durra, S. Karumbayaram, M. Mehrabi, C. J. Aros, S. D. Ahadome, D. W. Shia, K. Chung, J. M. Sandlin, K. F. Darmawan, K. V. Bhatt, C. C. Manze, M. K. Paul, D. C. Wilkinson, W. Yan, A. T. Clark, T. M. Rickabaugh, W. D. Wallace, T. G. Graeber, R. Damoiseaux, B. N. Gomperts, Modeling progressive fibrosis with pluripotent stem cells identifies an anti-fibrotic small molecule. Cell Rep. 29, 3488–3505.e9 (2019). 31825831 10.1016/j.celrep.2019.11.019 PMC6927560

[58] C. Venter, C. Niesler, A triple co-culture method to investigate the effect of macrophages and fibroblasts on myoblast proliferation and migration. Biotechniques 64, 52–58 (2018). 29571282 10.2144/btn-2017-0100

