# Supplementary material for: Modeling mechanical activation of macrophages during pulmonary fibrogenesis for targeted anti-fibrosis therapy
Source: Sci Adv. 2024 Mar 29;10(13):eadj9559. doi: 10.1126/sciadv.adj9559 (PMC10980276; doi:10.1126/sciadv.adj9559)
Supplement: Supplementary file 1 — Figs. S1 to S13 Table S1 [file sciadv.adj9559_sm.pdf]

Science Advances
AAAS

Supplementary Materials for

Modeling mechanical activation of macrophages during pulmonary fibrogenesis for targeted anti-fibrosis therapy

Ying Xu *et al.*

Corresponding author: Ruogang Zhao, rgzhao@buffalo.edu

Sci. Adv. **10**, eadj9559 (2024)
DOI: 10.1126/sciadv.adj9559

This PDF file includes:

Figs. S1 to S13
Table S1

Fig. S1.

(A) H&E staining of a portion of fibrotic lung tissue. (B) Confocal fluorescence images of an adjacent tissue slice of the sample shown in (A). Myofibroblast marker α-SMA (green), macrophage marker CD206 (red) and the merged channels with cell nuclei stained with 4′,6-diamidino-2-phenylindole (DAPI)(blue). Scale bar, 50 µm.

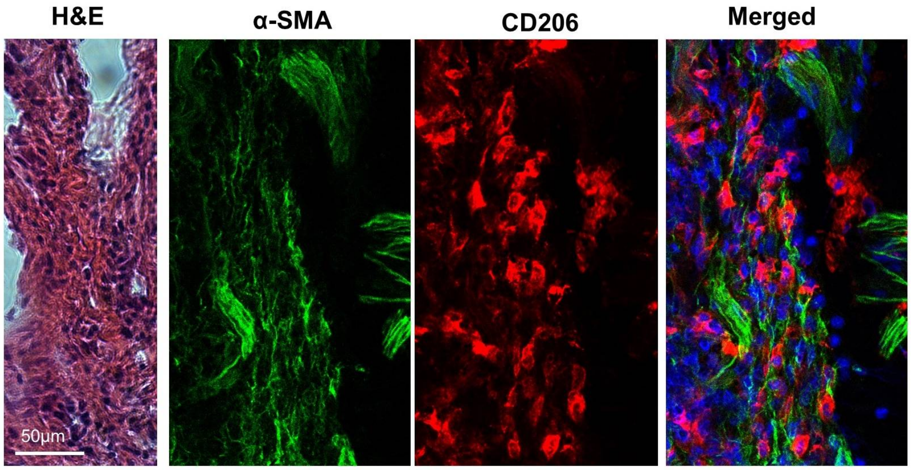

Fig. S2.

(A, B, C) Phase contrast images of fibroblast and macrophage co-cultured microtissues. (D, E, F) Confocal reflectance images of collagen fibers (cyan), and fluorescence images of F-actin (green), CD206 (red) and merged channels.

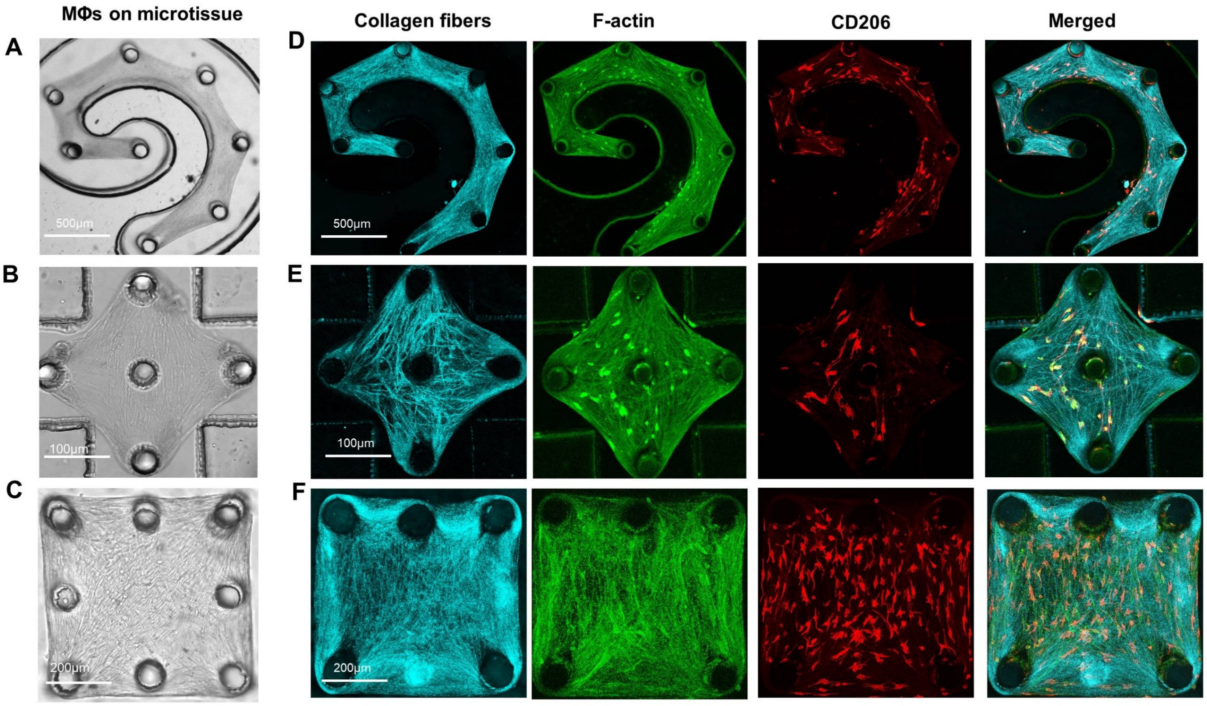

Fig. S3.

(A) Fluorescence image of fibroblast and macrophage co-cultured spiral microtissue, with nuclei stained in blue and integrin β2 (CD18) in yellow. (C) Fluorescence image of M2a macrophage monoculture on PDMS substrate (4 MPa). (B, D) Enlarged view of the boxed areas in (A, C) featuring integrin β2 (CD18) in yellow and nuclei in blue.

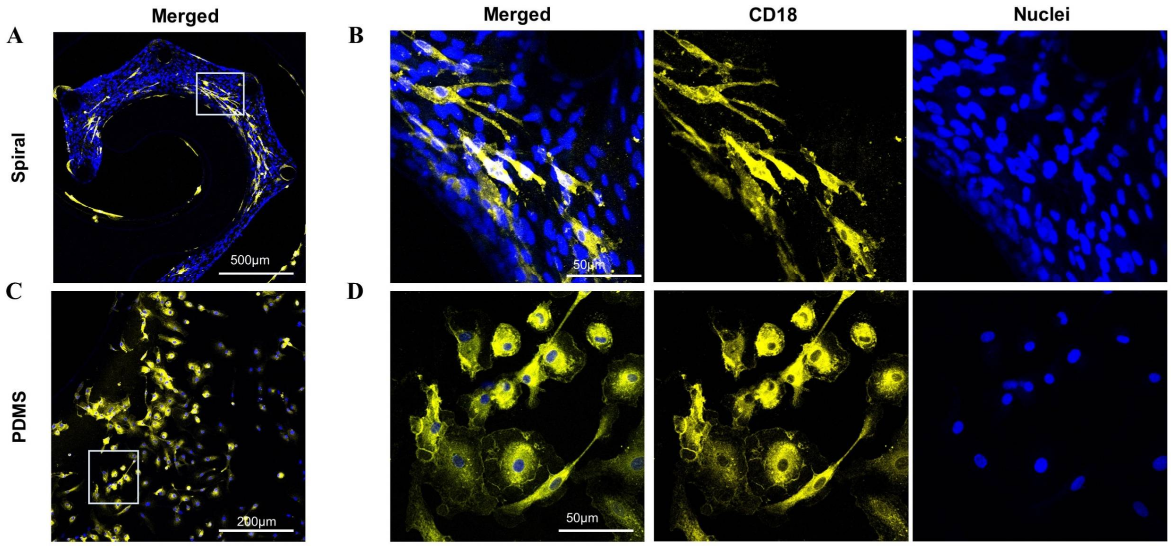

Fig. S4.

The co-culture of monocytes with NHLF populated square-shaped microtissue. Immunofluorescence images of nuclei (blue), monocytes (cyan cell tracker dye), CD206 stain (red) and α-SMA stain (green) at day 3 after adding monocyte to the microtissue. The level of CD206 and α-SMA expression is negligible.

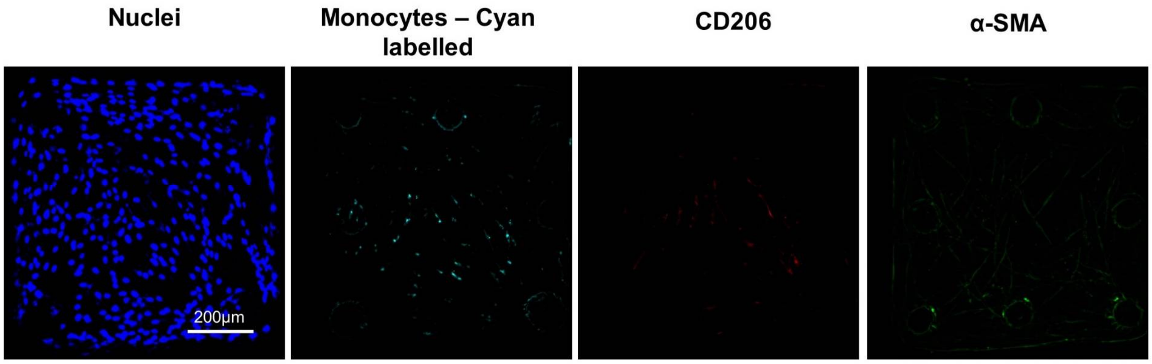

Fig. S5.

Monocytes (A) or M2 macrophages (B) seeded in fibroblast-free collagen microtissues. Monocytes or M2 macrophages were mixed with collagen type I and seeded in squared-shaped microdevice in the same manner as the fibroblast-populated microtissue. The images, from left to right, depict phase contrast images of monocytes or macrophages embedded in collagen in microwells, nuclei in blue, CD206 in red, fibronectin in green, and merged channels. There was no collagen gel compaction in both cases, suggesting the lack of cell generated contractile force. Monocytes were stained negative for fibronectin, but macrophages were stained positive for fibronectin.

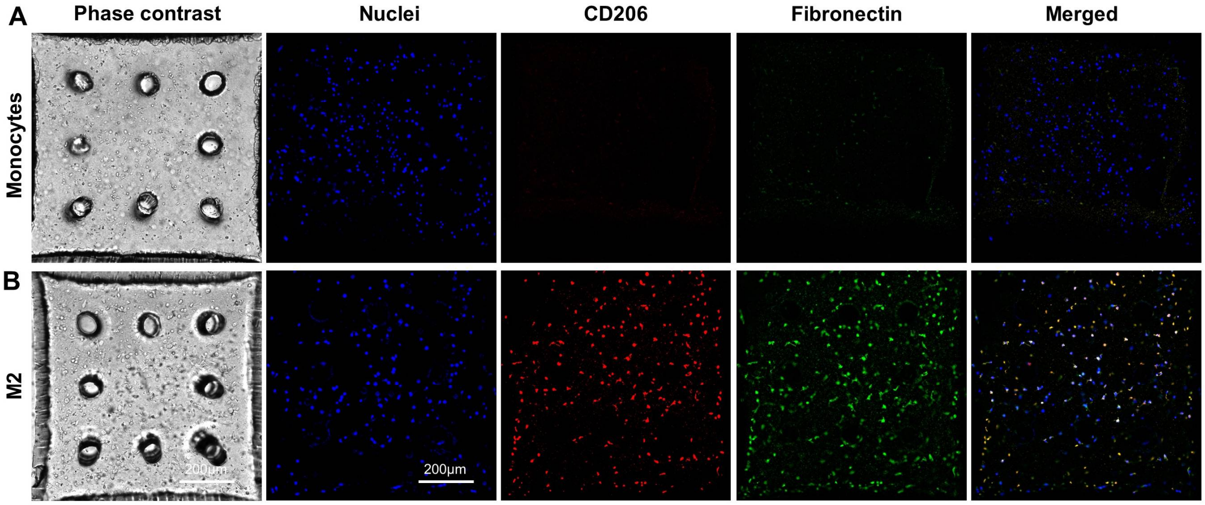

Fig. S6

Representative immunofluorescence images of co-cultured macrophages and NHLFs in microtissue model without PFD treatment (A), and with pretreatment (B), preventative (C) and therapeutic (D) treatment of PFD. The images, from left to right, show merged channels, nuclei in blue, CD206 in red, α-SMA in green, and TGF-β in yellow.

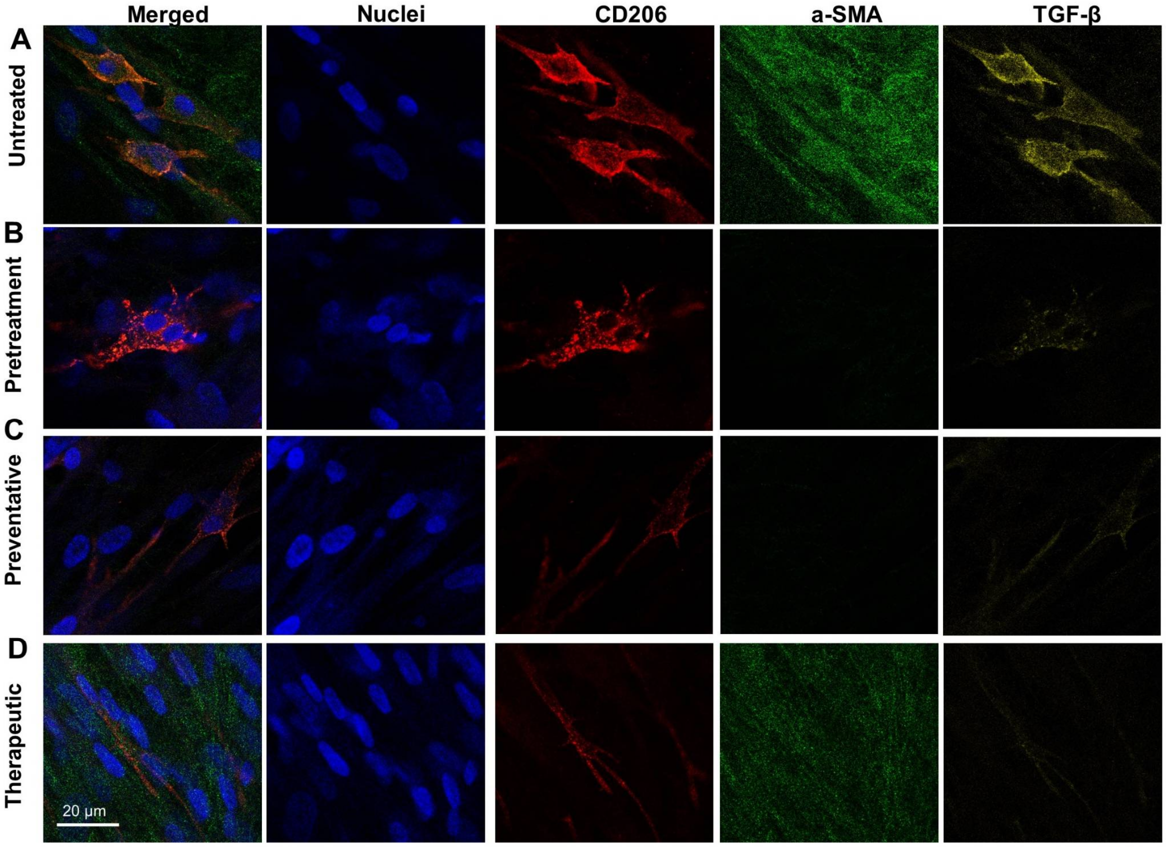

Fig. S7.

(A) Immunofluorescence images of NHLFs subjected to various treatments, including macrophage base medium (untreated), 5ng/ml TGF-β, M2a macrophage direct co-culture, non-heat activated (naïve) M2a macrophage culture supernatant, and heat-activated M2a macrophage culture supernatant. NHLFs were seeded on plastic petri-dish. Fluorescence channels include nuclei (in blue), α-SMA (in green), and CD206 (in red). (B) The fluorescence intensity of α-SMA was quantified for each of the treatment conditions.

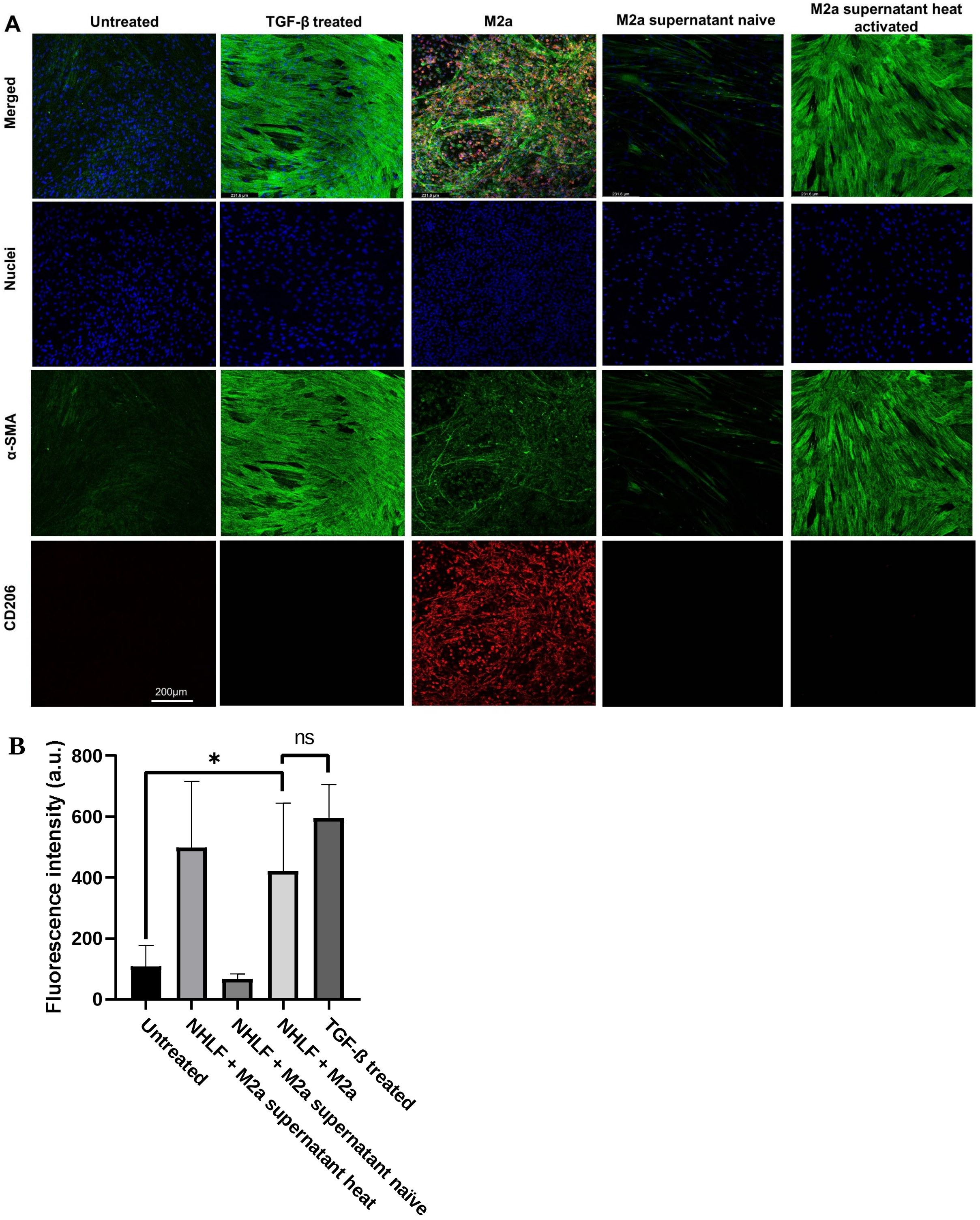

Fig. S8.

Representative immunofluorescence images of M2a macrophage and fibroblast co-cultured microtissue without PFD treatment (A), and under pretreatment (B), preventative (C) and therapeutic (D) treatment of PFD at 1000 µg/ml. The images, from left to right, show collagen fibers in cyan, fibronectin in green and CD206 in red.

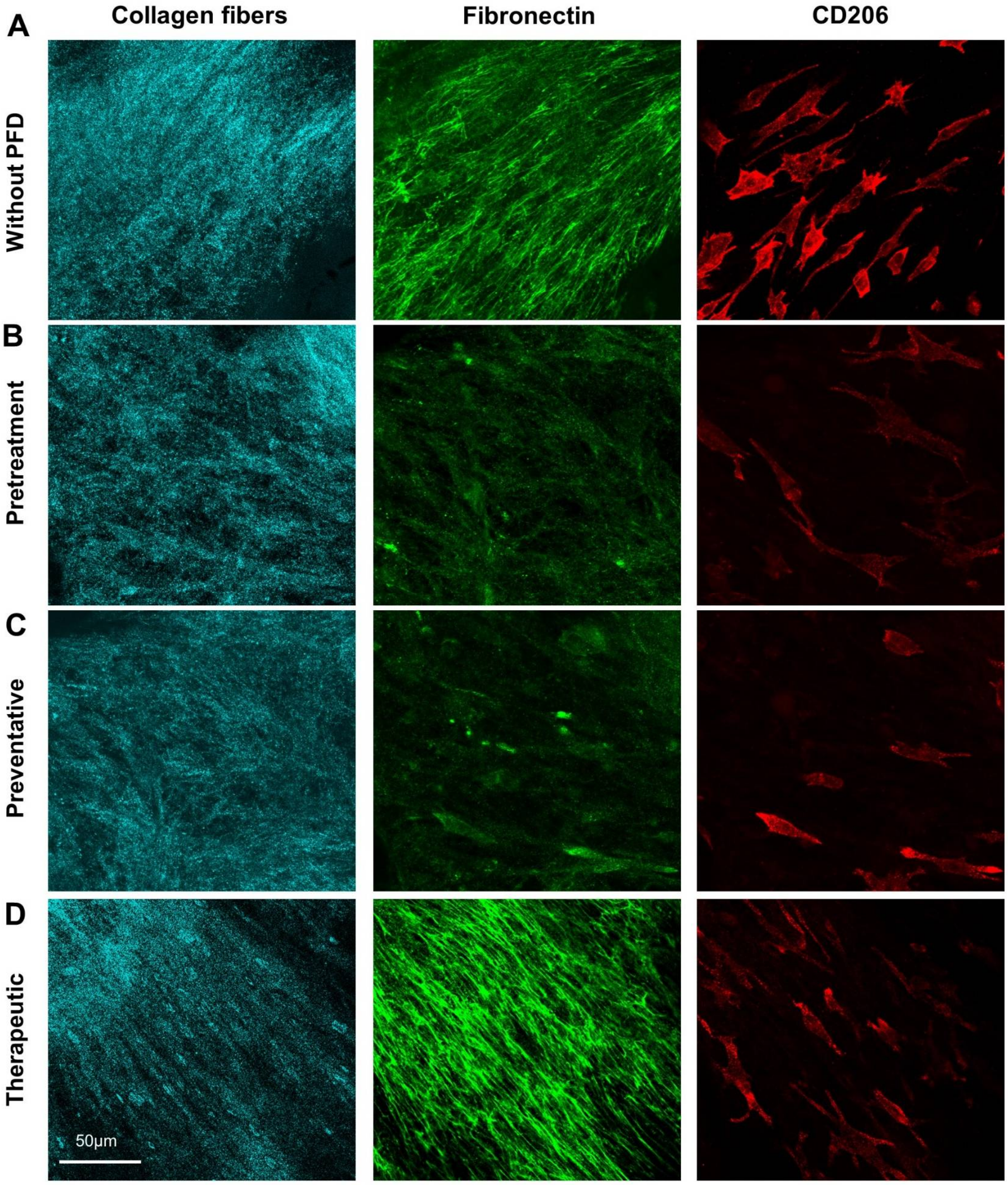

Fig. S9.

A representative standard curve for active TGF-β measurement using TMLC reporter cells. Luminescence measurements were converted into ng/ml of TGF-β by establishing standard curves performed with known concentrations of active TGF-β.

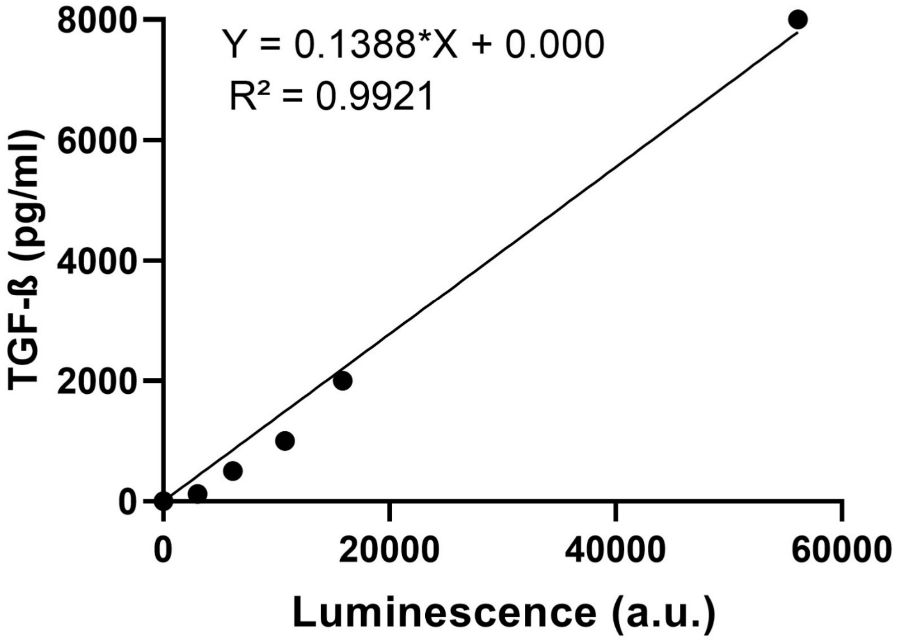

Fig. S10.

Effects of Pirfenidone (PFD) on the polarization of human PBMC derived monocytes towards the M2 phenotype. (A) Immunofluorescence staining of the M2 phenotypic marker CD206 (red) in monocytes pretreated with PFD for 24hs followed by the stimulation with or without IL-4 (20 ng/ml) + IL-13 (20 ng/ml) for additional 24hs. CD206 signal is overlaid with nuclei stain (blue). PFD inhibited the polarization of monocyte to M2 macrophages but did not cause significant macrophage loss. (B) Fluorescent intensity of CD206 in macrophages treated without or with PFD. (C) The concentrations of active TGF-β in cell culture supernatant of different culture conditions. Data are means ± SD from at least five experiments. *p<0.05 and ***p<0.01, when compared with the untreated group. #p<0.05 and ##p<0.01, when compared with the IL-4 + IL-13 stimulated group.

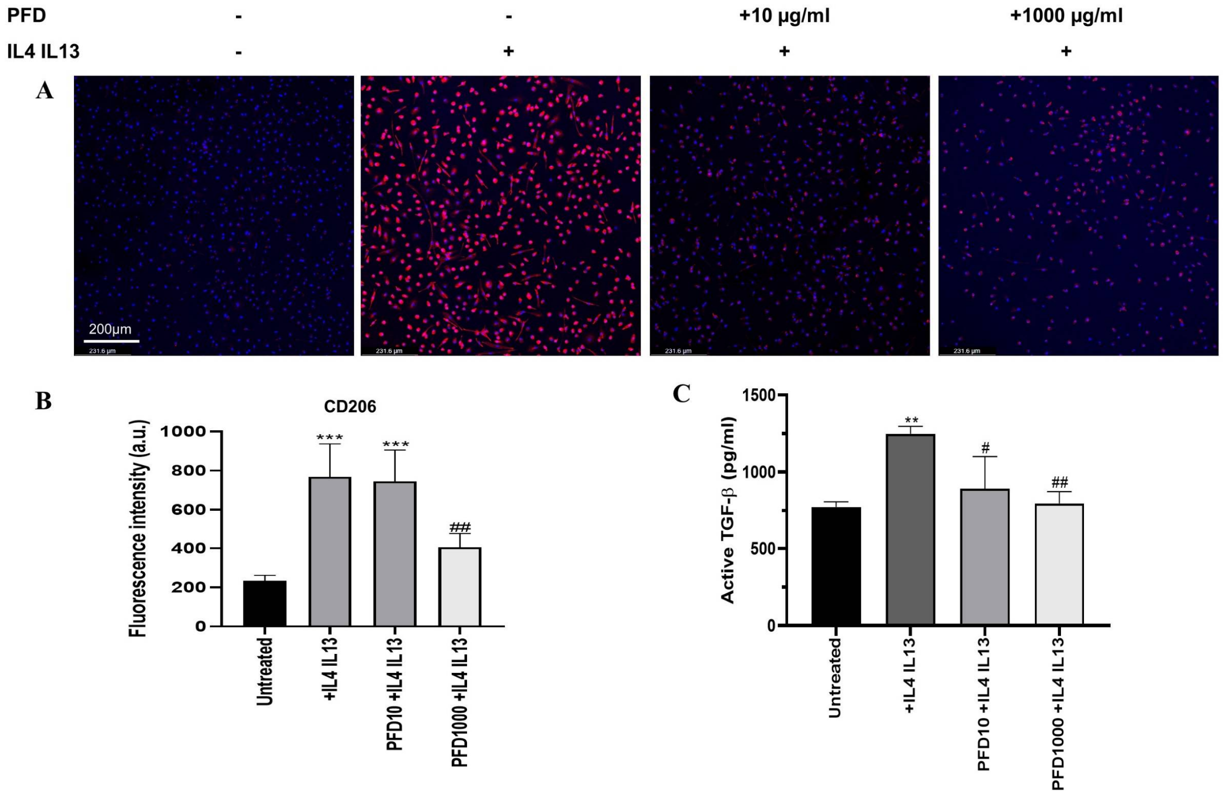

Fig. S11.

Phase contrast images of macrophages attached to regular and super soft PDMS substrates. PBMC monocytes were cultured on plastic dish and either treated or non-treated with pirfenidone for 24 hours, followed by treatment with IL4 and IL13 for additional 24 hours. Polarized macrophages were then detached from the original plastic substrate and seeded onto PDMS substrate and the number of adhered macrophages at day 3 were measured. (A, C) Macrophages without pirfenidone treatment on super soft and regular PDMS substrate, (B, D) macrophages treated with pirfenidone on super soft and regular PDMS. (E) The number of adherent macrophages on different substrates with or without PFD treatments. PFD caused a substrate stiffness dependent inhibition of the macrophage adhesion on PDMS substrate.

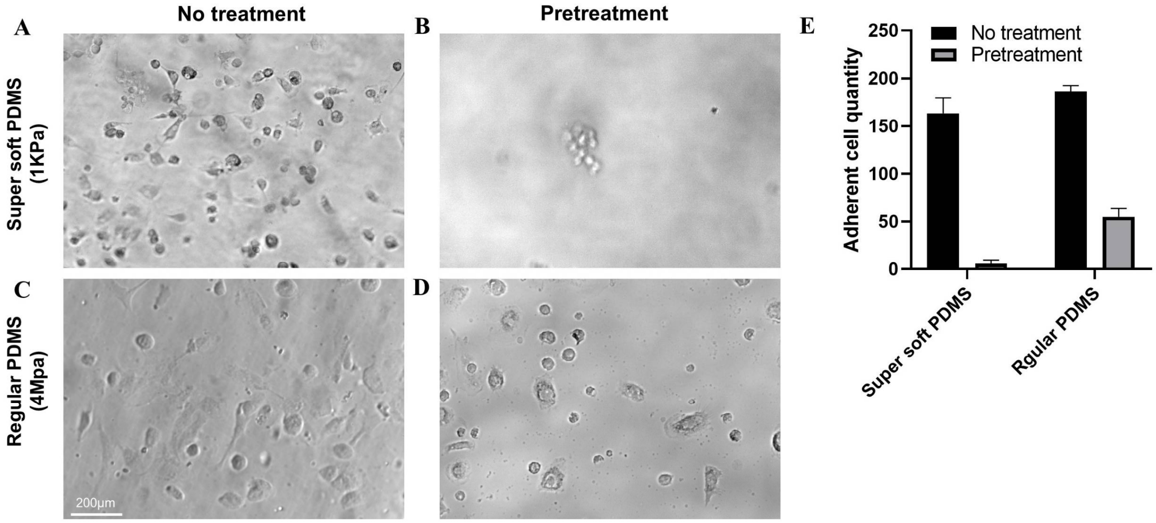

Fig. S12.

Representative immunofluorescence images of CD206 and ROCK2 in monocultured macrophages without PFD treatment (A), and under 10μg/ml PFD (B) or 1000μg/ml PFD (C) treatments. Macrophages were seeded on PDMS with stiffness of 4 MPa and stained for Nuclei (blue), CD206 (red) and ROCK2 (yellow) from top to bottom. (D) Fluorescent intensity of ROCK2 in monoculture macrophages treated without or with PFD. (E) The concentrations of active TGF-β in macrophage culture supernatant under different treatment conditions. Data are means ± SD from at least five experiments. *p<0.05 and ***p<0.01, when compared with the untreated group.

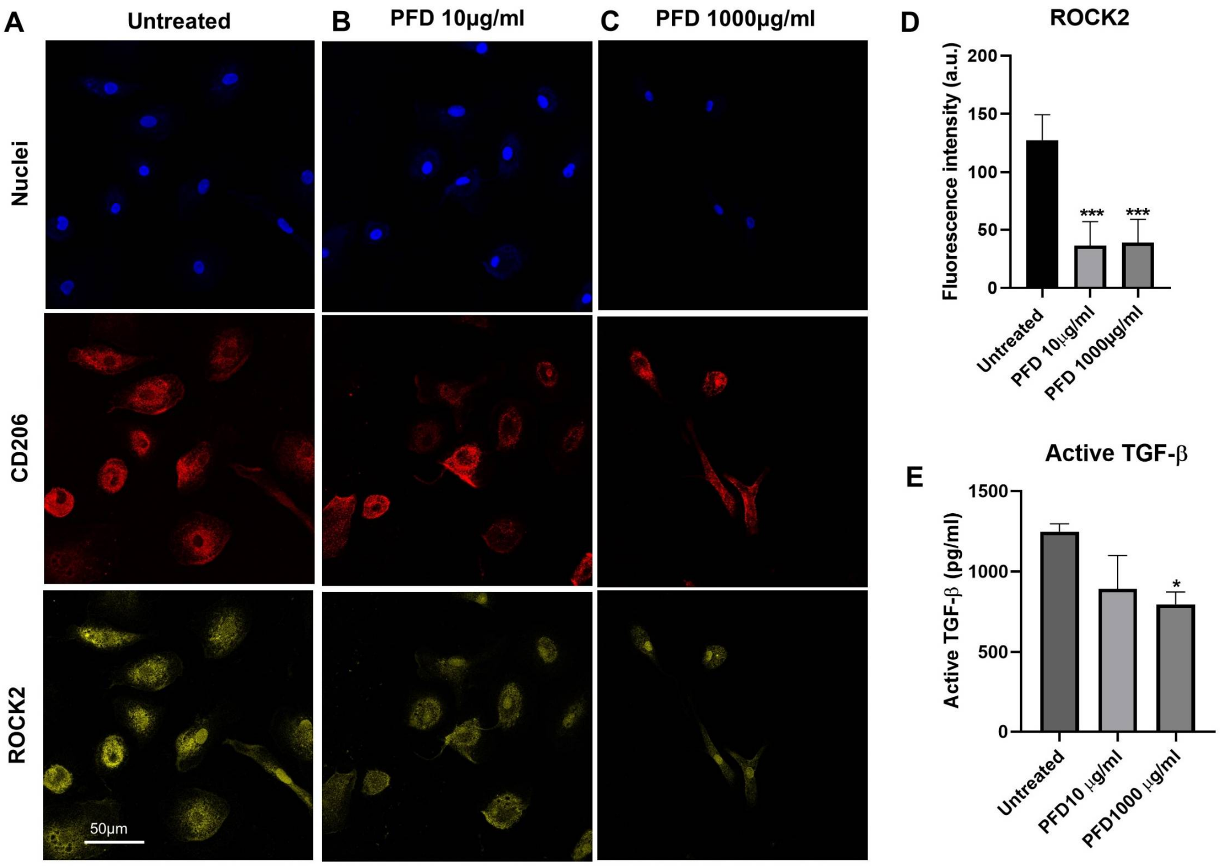

Fig. S13.

(A) Representative immunofluorescence images of CD206, CD 18 and CD 11b in monocultured macrophages under PFD or ROCK2 inhibitor KD025 treatments. Cells were seeded on glass and stained for CD11b (green), CD18 (yellow) and CD206 (red) from top to bottom. Fluorescent intensity of CD11b (B) and CD18 (C) in macrophages treated with PFD or KD025. Data are means ± SD from at least five experiments. *p<0.05 and ***p<0.01, when compared with the untreated group.

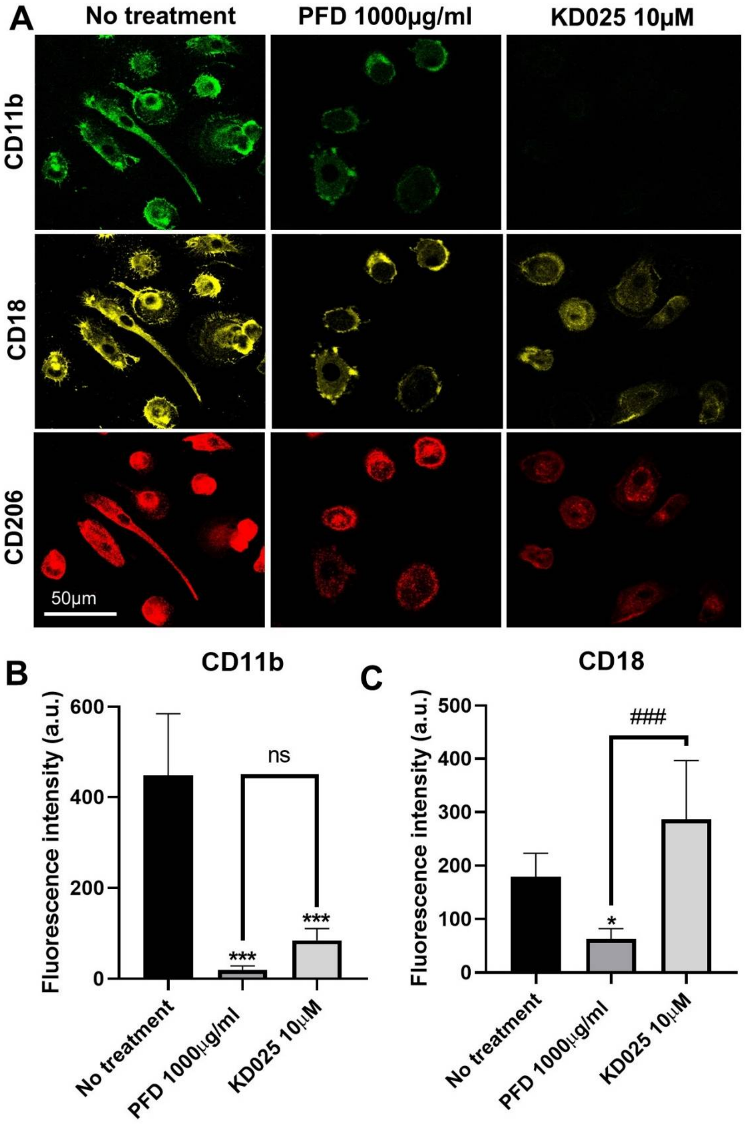

Table. S1.

Total values of microtissue contractile forces.

**Fibroblast only microtissue**	**Untreated**				
	Repeats	1	2	3	Mean	SD
	Day 0	122.8	118.1	115.0	118.6	3.9
	Day 1	116.5	105.6	135.5	119.2	15.1
	Day 3	115.8	119.4	136.2	123.8	10.9
	**TGF-ß treated**					
	Repeats	1	2	3	Mean	SD
	Day 0	115	133	139	129.1	12.5
	Day 1	129	149	154	144.3	13.5
	Day 3	131	143	167	147.1	18.4
**Co-culture of Fibroblasts and Macrophages**	**Untreated**					
	Repeats	1	2	3	Mean	SD
	Day 0	125	114	127	121.9	6.9
	Day 1	142	126	133	133.9	7.8
	Day 3	146	143	140	143.1	2.9
	**Pretreatment (PFD 10µg/ml)**					
	Repeats	1	2	3	Mean	SD
	Day 0	106.3	107.1	93.2	102.2	7.8
	Day 1	107.2	108.5	95.5	103.7	7.2
	Day 3	108.4	96.0	92.5	98.9	8.3
	**Pretreatment (PFD 1000µg/ml)**					
	Repeats	1	2	3	Mean	SD
	Day 0	135	129	112	125.0	11.9
	Day 1	136	120	122	126.2	8.6
	Day 3	123	131	121	124.9	4.9
	**Preventative (PFD 10µg/ml)**					
	Repeats	1	2	3	Mean	SD
	Day 0	135.0	100.9	101.4	112.4	19.6
	Day 1	135.9	102.3	108.4	115.5	17.9
	Day 3	137.1	95.5	94.6	109.1	24.3
	**Preventative (PFD 1000µg/ml)**					
	Repeats	1	2	3	Mean	SD
	Day 0	118.0	111.9	103.6	111.2	7.2
	Day 1	104.2	106.4	101.8	104.1	2.3
	Day 3	103.6	97.3	98.4	99.8	3.4
	**Untreated**					
	Repeats	1	2	3	Mean	SD
	Day 0	105.2	106.8	108.0	106.7	1.4
	Day 1	117.1	116.9	117.2	117.1	0.2
	Day 3	119.4	124.2	128.5	124.0	4.6
	Day 6	153.1	156.8	175.3	161.7	
	**Therapeutic (PFD 1000µg/ml)**					
	Repeats	1	2	3	Mean	SD
	Day 0	112.8	96.5	88.2	99.2	12.5
	Day 1	138.5	110.1	107.3	118.6	17.3
	Day 3	141.2	119.6	120.7	127.2	12.2
	Day 6	151.4	138.0	169.1	152.9	15.6
